# A scoping review of mental health prevention and intervention initiatives for infants and preschoolers at risk for socio-emotional difficulties

**DOI:** 10.1186/s13643-019-1043-3

**Published:** 2019-07-23

**Authors:** Alan McLuckie, Ashley L. Landers, Janet A. Curran, Robin Cann, Domenica H. Carrese, Alicia Nolan, Kim Corrigan, Normand J. Carrey

**Affiliations:** 1 University of Calgary, Calgary, Canada; 2 Department of Human Development, Virginia Polytechnic Institute & State University, 7054 Haycock Road, Falls Church, VA 22043 USA; 3 Dalhousie University, Halifax, Canada

## Abstract

**Background:**

Infant mental health has emerged as a unique area of practice and research distinguished from child and youth sub-specialties by its advocacy for a relational practice framework with an emphasis on parents/caregivers being integral to assessment, treatment, and prevention initiatives. A diverse array of initiatives offered across a broad spectrum of delivery methods is available to clinicians. However, to date, a large-scale mapping of the research evidence regarding these interventions has yet to be completed to help inform clinician’s decisions regarding the best approaches for their clients. To address this knowledge gap, this study aimed to report on the landscape of research pertaining to mental health interventions for infants and preschoolers (0–5 years), and their families at risk for socio-emotional difficulties and negative developmental outcomes.

**Method:**

A scoping review methodology was used to conduct a large-scale mapping of the intervention research pertaining to infants and preschoolers (0–5) at risk for socio-emotional difficulties. We searched MEDLINE, PsycINFO, EMBASE, Web of Science, The Cochrane Library, CINAHL, LILACS, ProQuest Nursing & Allied Health Source, World Cat, and ClinicalTrials.gov, from inception to December 31, 2012. We extracted information regarding publication date, geographical location, study design, level of risk, population, key intervention mechanism, and outcome measures.

**Results:**

We identified 533 potential studies from 1233 title and abstracts after the first round of screening. Full text article review in the second round of screening resulted in a total of 162 included articles for the final analysis. Results indicated that over 50% of interventions evaluated were randomized controlled trials conducted in Westernized countries. Most studies could be subdivided by level of risk within a preventative public health framework including universal, selected, indicated, and direct treatment for children formally diagnosed with a mental disorder. Risk factors experienced by children and their families were heterogeneously defined and numerous outcome measures across included studies. The results of this study are limited to the last search date of 2012.

**Conclusions:**

Key intervention mechanisms spanned a range of approaches including parenting groups, dyadic, in-home, cognitive-behavioral therapy, and day care-based interventions. The findings are discussed in terms of implications for broad trends and gaps in research and policy for this population.

**Electronic supplementary material:**

The online version of this article (10.1186/s13643-019-1043-3) contains supplementary material, which is available to authorized users.

## Background

Infant mental health (IMH) has emerged as a unique area of practice and research distinguished from child and youth sub-specialties by its advocacy for a relational practice framework with an emphasis on parents/caregivers being integral to assessment, treatment, and prevention initiatives [1]. Interest in IMH was driven initially by clinical research in the areas of childhood attachment, socialization, and development [2–6] and by large-scale US-based social initiatives of the 1960s and 1980s, such as *Head Start*, that focused public attention on the merits of prevention-based programs to invest in infant well-being. Attachment theorists and proponents of pre-school programming for at-risk families [7] have helped to drive research forward and solidify the field’s knowledge-base as to the effectiveness of various programs for positive developmental trajectories. In addition, advances in assessment and treatment as well as longitudinal studies over the last 30 years have demonstrated that psychopathology which results from the combination of environmental and genetic risk factors can be reliably identified in preschoolers [1]. Therefore, infancy and early childhood are key developmental periods during which precursors to significant and lasting mental disorders may emerge, while at the same time representing key timeframes for prevention and early intervention.

The study of risk factors for early-onset mental health issues has evolved to recognize the dynamic interaction between individual genetics and temperament with the child’s environment in shaping developmental outcomes. The absence of secure parent-child attachment and the presence of adverse or traumatic experiences, especially during sensitive and/or critical periods of development are believed to be key to the onset and continuance of early childhood emotional and/or behavioral issues [8, 9]. This age group is also considered to be most at risk for experiencing abuse and/or neglect [10, 11]. Other specific risk factors associated with the presence of mental health difficulties in infants and preschool children include maternal depression, parental substance abuse, family violence, limited parental education, poverty, and neighborhood safety issues [12, 13].

Early childhood behavioral problems, including disruptive behaviors, oppositional defiance, and/or aggression, represent one of the most common referrals for mental health intervention [14, 15] with anxiety disorders being the second most frequent concern [16, 17]. Prevalence rates of preschool psychopathology vary between 7 and 28% [18] and show developmental continuity with later childhood and adolescent psychopathology [14]. In Early Childhood Education (ECE) settings, estimates suggest that as many as 30% of children require special attention within their programs due to emotional regulation or behavioral difficulties [19]. While some risk factors may be transient and not affect the child’s developmental trajectory, the cumulative interaction of environmental and genetic risk factors in some children may lead to enduring problems without intervention [1].

Research on the effectiveness of infant and preschool psychosocial interventions and prevention initiatives has grown significantly since the emergence of IMH as a distinct sub-speciality in the late 1970s. Treatment initiatives for children diagnosed with mental disorders and/or those with emergent difficulties typically involve attempts to alter the child’s deviant behavior or emotional dysregulation, and/or negative interactions within the infant-caregiver dyad, either through direct work with this dyad or indirectly through parent education programming. Regardless, in most cases, treatment for IMH issues considers the parent-child relationship as the principal mechanism for change. Although the parent-child relationship is typically the primary focus of assessment, intervention, and prevention initiatives, the functioning of the child’s broader contexts, such as family and community factors, are also viewed as important influences shaping developmental trajectories and are thus included in treatment interventions and prevention initiatives [1].

Due to the shifting influence of risk factors during this developmental period, conceptual frameworks for infant and child mental health programming must include both prevention and intervention initiatives. One such framework, commonly used in public health reporting [1, 20, 21], categorizes prevention initiatives according to the level of risk targeted by the intervention and/or the degree with which a particular health problem may be experienced by each social stratum within the population under examination. Initiatives are sub-classified as programs that are universal, selective, indicated, and direct treatment for children diagnosed with and/or experiencing mental disorders. Universal programs are offered to the broadest range of infants, preschoolers, and families, and are considered beneficial regardless of the presence of unique risk factors, problems, or need for professional intervention (e.g., state-wide/provincial subsidized high quality daycare). There is a growing body of selective prevention initiatives targeting groups deemed at-risk for future developmental outcomes. The best researched of these initiatives is the federally funded US-based *Head Start* programs, which provide comprehensive early childhood education, health care, nutrition, and parenting programming for groups considered at-risk due to systemic poverty. Indicated programs are provided to children demonstrating sub-clinical problems of recognizable difficulties and/or developmental issues that if left unaddressed, will likely develop into full-scale syndromes or disorders requiring professional treatment interventions [22, 23]. A further category is direct treatments by mental health professionals to children diagnosed with mental disorders as defined by mostly categorical criteria [1].

This diverse array of interventions offered across a broad spectrum of delivery models, raises several questions including: What populations are serviced by the various approaches? What are the aims of these interventions? In what settings are these interventions conducted? What research methods, including measurement tools and/or data-collection are used? To date, a large-scale mapping of the landscape of the research regarding infant and pre-school early interventions and prevention initiatives has not yet been conducted. Therefore, in order to answer these questions and determine the composition of the research landscape, a scoping review was conducted [24, 25]. A scoping review is a process of mapping key concepts, the main sources, and types of evidence available as well as gaps in a research area especially where an area is complex or not previously reviewed in a comprehensive manner [26]. Our objective therefore is to determine the scope of the mental health intervention and prevention research pertaining to infants and preschoolers (0–5 years), and their families at-risk for socio-emotional difficulties and negative developmental outcomes.

## Methods

The methods for this scoping review were informed by the six-step procedure outlined by Daudt, van Mossel, and Scott [27], an extension of Arksey and O’Malley’s [24] approach and reported following PRISMA statement extension for scoping reviews [28] (Additional file 1). Our team’s “interpretation of how the consultation is achieved” [27, p. 7] was to adopt an integrated knowledge translation and exchange (iKTE) approach, which involved consulting with our knowledge users (KU) through each step of the review procedure.

For the initial step, to define the research questions, we worked closely with the project’s KU group, comprised of professionals representing social services, tertiary health care, public health, and early childhood education. Together, we refined the parameters of our scoping review by posing three research questions: (a) What intervention and/or prevention programs currently exist for children ages 0–5 at risk for mental health difficulties, and their caregivers and families? (b) What populations participated in these interventions and preventions programs? (c) How are these programs evaluated? In order to transform these questions into searchable queries consistent with research database requirements, we worked with an information scientist to define our key constructs including “at risk for mental health,” “intervention and prevention programs,” “caregivers,” and “families.” We defined “at risk for mental health” as an infant or toddler displaying deviant behavior and/or emotional patterns, interpersonal difficulties, or those demonstrating psychopathology, or diagnosed with a disorder, or exposed to known risk factors for the same, including maternal mental health issues, caregiver substance abuse, poverty, and residing in a community with safety concerns. Caregivers were defined as a parent and/or a primary caregiver responsible for providing day-to-day care and the emotional developmental needs of the child. Family was defined as including the primary caregiver/parent as well as siblings and relatives or those considered family residing in same family home and/or taking on responsibility for caregiving of the preschooler. Intervention and prevention programs were defined as any or all models, services, strategies, and/or techniques provided by professional, para-professional, or lay person purported to address, remediate, accommodate, offset, or reduce the chances of onset or continuance for mental health difficulties or disorders, behavioral or emotional deviance, or developmental issues.

In the second stage of our review, we endeavored to identify all relevant studies. Conducted in collaboration with our information scientist, we engaged a systematic search of all relevant online research databases, as well as used snowball search methods and reference tracking (i.e., checking reference lists of included sources and checking database alerts) to identify additional articles. Database searches included MEDLINE, PsycINFO, EMBASE, Educational Resources Information Centre (ERIC), Clinicaltrials.gov, Cumulative Index to Nursing and Allied Health Literature (CINAHL), The Cochrane Library, including the Cochrane Central Register of Controlled Trials (CENTRAL), ProQuest Nursing & Allied Health Source, Latin American and Caribbean Literature on the Health Sciences (LILACS), Web of Science, and World Cat. Key search terms, including synonyms and medical subject headings (MeSH terms), were entered into these databases in systematic manner by a library scientist. Search parameters for dates of publication included all eligible studies published from inception through December 31 of 2012. Additional file 2 contains the search terms employed for Medline Ovid. Medline was searched first because it was considered the most relevant for our study’s objectives. Terms were subsequently modified as required for each search of the various databases engaged in the current review. We also conducted a search of the gray literature via the following portals: Canadian Institutes of Health Research; Centre of Excellence for Early Childhood Development; CMA Infobase; Institute for Research on Public Policy; International Network for Early Child Development; International Organization for Early Intervention; National Guidelines Clearinghouse; Offord Centre for Children at Risk; World Health Organization; World Association for Infant Mental Health. We also conducted a hand search of the table of contents for the *Infant Mental Health Journal*, *Child Development*, the *Journal of the American Academy of Child and Adolescent Psychiatry*, and the *Journal of Child Psychology and Psychiatry* for articles published between 2008 and 2012.

The third phase of the review, study selection, began with two reviewers independently screening titles and abstracts against specified inclusion and exclusion criteria to determine suitability for inclusion in the study. Duplicate citations were removed automatically from our *RefWorks* library. During the next stage of screening, the same reviewers independently screened the full text articles deemed relevant in the first stage of citation screening. Disagreements regarding study inclusion during the title/abstract or full text review processes were resolved by a senior member of the research team (N.C.) if consensus could not be achieved through discussion. This process was required for 20 articles during the title/abstract review phase for which consensus was not achieved, which represents an inter-rater reliability for inclusion of 98.4%. Consistent with our published scoping review research protocol [29], we considered for inclusion any/all studies demonstrating a clearly articulated and research methodology, including quantitative (i.e., randomized controlled trials, quasi-experimental designs, single group pre-post with and without follow-up, case-control), qualitative (case studies, phenomenology, ethnography, grounded theory), or mixed methodologies. We excluded review articles or studies presenting filtered information (i.e., systematic reviews, scoping reviews, evidence syntheses, narrative reviews, qualitative syntheses). Qualitative studies were also included where rigorous qualitative methodologies were clearly reported, including those adhering to a case study methodology. However, papers offering clinical scenarios, vignettes, case descriptions, or clinical examples that failed to report a research methods consistent with qualitative case study methods were excluded. Specific a priori inclusion criteria required that all included studies were (a) primarily related to the examination of and/or reporting on psychosocial interventions involving at-risk preschooler; (b) parents demonstrating risk factors or adversity (e.g., depression, substance abuse), or the parent-child relationship demonstrating risk factors or adversity (e.g., negative parenting practices, abuse); (c) a reliable assessment of baseline mental health diagnosis or risk factors and at least one child-related mental health outcome; (d) a child sample/population at risk for socio-emotional difficulties (e.g., anxiety, depression, aggression); and (e) a child sample/population 0–5 years of age, or a sample/population of parents of children 0–5 years of age, or the sample/population being reported upon is the long-term follow-up of a population that was 0–5 years of age in the original study. Studies were excluded if they (a) primarily examined autism (autism spectrum disorder) or developmental disorders including intellectual or emotional impairment (i.e., mental retardation, severe fetal alcohol syndrome), language/communication disorders (i.e., receptive, expressive disorders); (b) studies focusing primarily on assessment with no intervention component and no outcome measures; and (c) generic parenting groups not targeting change in the parent-child relationship and/or the child’s current or future socio-emotional/developmental outcomes.

In the fourth stage of the review, we charted data, first by developing an extraction tool used in combination with an Excel spreadsheet to systematically record pertinent information for included studies. Categories included in the data extraction tool and spreadsheet were as follows: (a) publication year, (b) author, (c) location of study (country), (d) setting for study (i.e., clinic, community, home), (e) population targeted, (f) age of participants, (g) type of intervention, (h) outcome measures used, (i) study outcomes, (j) duration of intervention, (k) number of participants, (l) cost of program, (m) professional background of treatment provider, (n) methodology for study, and (o) whether the study was a replication. Consistent with Levac and colleagues’ [25] suggestion, two senior members (N.C. and A.M.) of the research team applied the extraction tool to the same five studies to determine the inter-agreement of our processes. Following this process, two reviewers used this tool to independently extract the data from each study included in this scoping review with discussions at regular intervals to ensure coding accuracy. As an extra degree of inter-agreement, a senior member of the research team (A.M.) reviewed 10% of completed extractions and compared these to the original full text articles to further verify the inter-agreement of our extraction process. No concerns were found with the extraction process.

The fifth stage of the review, known as the collating, summarizing, and reporting phase, was the most intensive stage of the study where we analyzed the data, reported the results, and applied meaning to these results [25]. To efficiently and effectively conduct the analysis, we examined the data-extraction chart corresponding to each study included in the review. Two members of the research team (A.M. & R.C.) analyzed this material to identify key themes and/or commonalities and differences between and across studies.

The sixth stage, the consultation process, involved an iKTE process whereby project researchers collaborated closely with project KUs in order to ensure the relevance and usefulness of the final product. Although this is described as the final stage in the review process, it actually occurred throughout each stage. We collaborated with our KU partners through face-to-face meetings, teleconferences, and email document exchange to refine the research questions, finalize terms for the search strategy, identify important data extraction elements, and interpret findings.

## Results

**Fig. 1:**
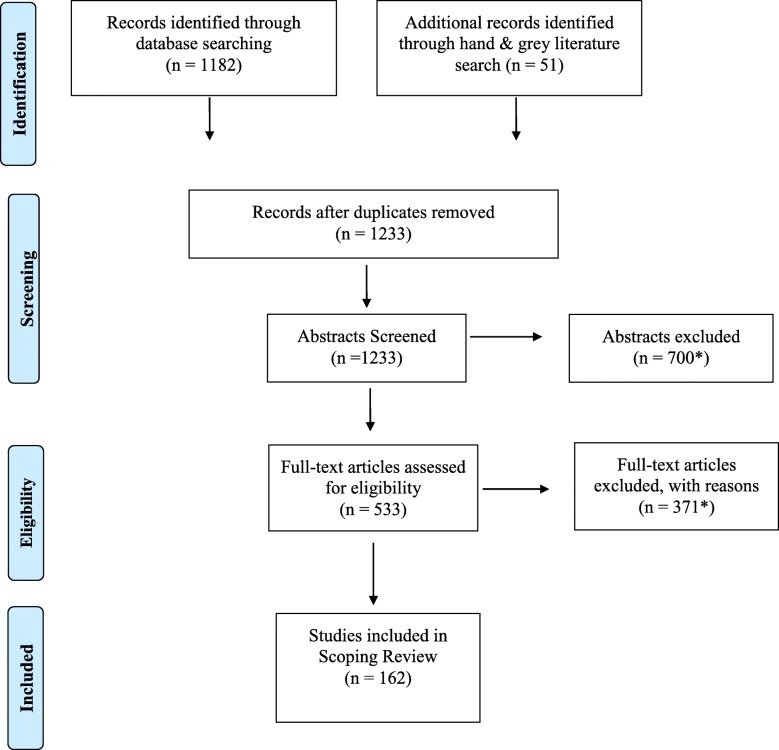
PRISMA diagram. Articles were excluded following abstract and full-text with a priori inclusion/exclusion criteria outlined in the manuscript [224]

We identified 533 potential studies from 1233 title and abstracts after the first round of screening. Full text article review in the second round of screening resulted in a total of 162 included articles for the final analysis. Figure 1 shows the study selection procedure in PRISMA format. A table presenting key extracted data from all 162 articles can be found in an online appendix (Additional file 3), which also includes key extracted data from 23 studies not referred to elsewhere in this manuscript.

### Timeline (dates) for research

**Fig. 2:**
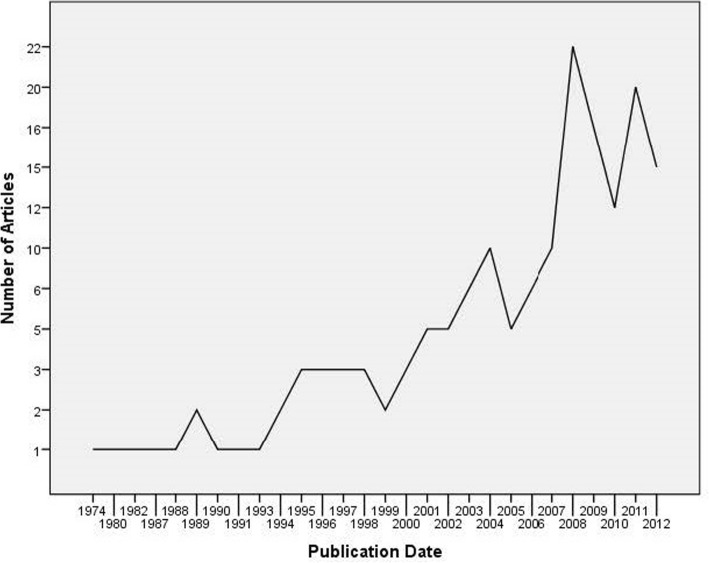
Infant mental health publications per year

The earliest identified study meeting our inclusion criteria was published in 1974 [30]. Little growth in IMH research occurred for the next 20 years with only one or two publications per year in the 1980s (*n* = 7) and only a few each year in the 1990s (*n* = 20). This trend changed at the turn of the century with a rise in publications rates between 2000 and 2006 (*n* = 40) and a spike in research (*n* = 94) from 2007 through 2012 (see Fig. 2 for the number of publications per year).

### Location (country) of research

Empirical studies on IMH initiatives have largely been driven by researchers from the USA (54%; 88) and Australia (17%; *n* = 28). Together, research from these two countries represented 72% of all studies included in the current review. Research was also conducted in the UK (*n* = 14), Canada (*n* = 6), the Netherlands (*n* = 6), Romania (*n* = 3), Sweden (*n* = 3), China (*n* = 2), Germany (*n* = 2), Japan (*n* = 2), South Africa (*n* = 2), Ireland (*n* = 1), Israel (*n* = 1), Pakistan (*n* = 1), Puerto Rico (*n* = 1), and Switzerland (*n* = 1). One study did not report country of origin.

### Study designs

A diverse range of research methods was employed within the studies included in this scoping review. The majority were randomized controlled trials (RCTs; 51%; *n* = 82) representing half of the total number of studies included in the current review. There were also nine (*n* = 9) long-term follow-ups on previously conducted RCTs, as well as five (*n* = 5) clustered RCTs, with one (*n* = 1) long-term follow-up. Two (*n* = 2) studies conducted latent transition analysis on previously conducted RCTs. Quasi-experimental designs (QED) in various form represented the next most common research methodology (23%; *n* = 37). Of these 37 QED studies, 20 were pre and post intervention comparisons, and ten were pre-post with follow-up conditions, three were QED repeat measures, and two could only be identified as quasi-experimental. Additional study designs included seven (*n* = 7) one-group pre-test post-test designs with follow-up and without follow-up (*n* = 8), four qualitative methods case study design (*n* = 4), one (*n* = 1) repeat measures design, two (*n* = 2) case control, one (*n* = 1) qualitative methods content analysis design, one (*n* = 1) prospective single group repeat measures designs, one (*n* = 1) prospective cohort design, as well as one (*n* = 1) mixed methods design (i.e., quantitative data via archival and survey methods and qualitative data via focus group and in-depth interviews).

### Level of intervention and population targeted

In the current scoping review, the characteristics of the majority of programs/interventions examined along with the populations they targeted (i.e., their research samples) aligned with a nested public health framework proposed by Haggerty and Mrazek [21] and others [20]. Using the child as the reference point for the intervention (i.e., the identified patient), the interventions/programs were organized into the four categories of (a) direct treatments for children with diagnosable mental disorders, (b) indicated prevention initiatives for children with sub-clinical problems of recognizable difficulties and/or developmental issues, (c) selective prevention programs that target children and/or families who are at high risk for mental and/or developmental problems, and (d) universal programs offered to children and families regardless of existing risk factors. The following section addresses these categories in order of prevalence amongst the included studies.

#### Selective prevention programs

The majority of programs and interventions described in research studies were considered to be at the selective level of intervention (58%; *n* = 94). These interventions were provided to populations deemed to be at-risk by experiencing broader structural factors such as poverty, familial risk factors (e.g., parental mental health issues), and temperamental factors (e.g., child sleep problems or behavioral difficulties). One study conducted by Kennedy, Rappee, and Edwards [31] could be considered to be both selective prevention and direct treatment, as the research sample targeted parental anxiety and the children included were diagnosed with mental disorders.

#### Indicated prevention programs

Indicated prevention programs were the second most common initiatives (26%; *n* = 42) identified in the included studies. These studies investigated interventions provided to children without formal diagnoses but who displayed behavior problems and/or elevated behavioral scores on screening tools. The study by Bor, Sanders, and Markie-Dadds was identified under this category because it could also be categorized with the selective prevention group due to their recruitment strategy “targeting disadvantaged families” ([32], p. 574). However, the decision was made to align this intervention with the indicated group because the study’s inclusion criteria required children to score in the elevated range for behavioral problems on the Eyberg Child Behavior Inventory [33]. Similar categorizations were made for other studies sharing similar recruitment/inclusion criteria that focused on the presence of subclinical child behaviour problems as well as the presence of sociodemographic and/or familial risk [34–45]. The Connell et al. [46] study was assigned an asterisk because it could also fit into two categories of intervention. This study’s sample conforms with both the indicated group because its recruitment targeted children with sub-clinical behavioral problems, as well as with the selective prevention group due to inclusion criteria related to risk factors such as poverty and family/parental history of mental disorder.

#### Universal and direct interventions

Universal interventions and direct treatment by a mental health professional for a diagnosed mental disorder were the least common within the current review. Universal interventions represented 7% (*n* = 12) of the included studies. Direct psychosocial interventions by a mental health professional for children diagnosed with a mental disorder represented 9% (*n* = 14) of the interventions. Children with a formal DSM diagnosis included oppositional defiant disorder (ODD), ADHD, ADHD with ODD, conduct disorder, anxiety, and major depressive disorders. Some studies did include autism spectrum disorder and global developmental delay but the sample sizes representing these categories in these studies were small. One of the studies, Abrahamse and colleagues [47], was identified with an asterisk because it could fit into the direct category, due to its sample being comprised of a subset of children diagnosed with a mental disorder (45.9% of the sample) or within the indicated group due to over half of its sample with subclinical disruptive behavioral issues. Similarly, in the study conducted by Levac and colleagues, only “some children” ([48], p. 81) met diagnostic criteria for conduct disorder and/or ADHD, while the remainder of the sample demonstrated subclinical aggressive behaviour patterns.

Throughout all levels of the prevention intervention framework, no consensus existed as to a common definition of “risk,” what factors were necessary and sufficient for risk to be actual versus theoretical, and agreement on how best to measure risk. We analyzed how risk was defined in the most populated prevention category (i.e., selective prevention). Children were considered “at-risk” due to elevated scores on behavioral measures, such as the ECBI [38–40, 49–51], parents were deemed “at-risk” via elevated scores on measures such as the Centre for Epidemiological Studies Depression Scale [52, 53], and families were considered “at-risk” due to elevated scores on measures such as the Family Stress Checklist [54, 55]. The child and/or family were also considered in some studies to be “at-risk” due to the presence of adverse family circumstances including teen pregnancy/teen mothers [56–58] or caregiver attachment issues [59–61]. The two most common adverse family conditions cited in the research were parental mental health and addictions issues [31, 53, 62–70], in particular maternal depression [52, 60, 63, 71–81] and low income or poverty [30, 34, 82–92]. Risk was also determined by the family residing in “at-risk” communities [93–96] or due to the child and/or family’s involvement in certain programs or agencies, typically used as a proxy for their “at-risk” status, for example, enrolment in early *Head Start* programs or similar [52, 62, 97–113] or involvement with child welfare services [114–118].

### Key mechanisms of interventions and principle settings of implementation

Embedded within each of the treatment interventions and prevention initiatives included in the current review lie mechanisms of therapeutic change considered central to achieving the desired outcomes. Interacting with contextual variables associated with the study’s settings and populations, these therapeutic mechanisms can be distilled into a few key mechanisms common across several of the interventions and prevention initiatives. Five key mechanisms were apparent in the current review including parent-education/skills training, dyadic parent-child relational interventions, interventions where home visitation was the central mechanism of change, pre-school/daycare-based interventions and programs, as well as cognitive behavioral therapy-based programs.

Initiatives and programs included in the current review were also conducted in a variety of settings such as clinic-based, community-based, home settings, or a combination of these. Only the key therapeutic mechanisms and the principal settings for the interventions/initiatives are reported and in the case of studies with comparison conditions (i.e., RCTs), only details pertaining to the intervention/treatment condition are reported.

Parent education programming and/or skills-based programming directed at the parent/caregiver/family typically to effect change in the child’s behavior were offered mostly within clinic-based settings focused on mental health interventions (*n* = 28) and community-settings, such as community centres and/or parenting-centres (*n* = 12). Some were offered in dual locations, including home and community-based initiatives, clinic and home, clinic and phone, school-based setting, school and community, clinic and community, a university/research setting, or at the service-user’s home.

Dyadic parent-child relational interventions were also offered most commonly in clinic-based settings (*n* = 26) but relative to parenting skills programs were provided more frequently within the service users home (*n* = 17). Dyadic-relational interventions were also offered in joint home and clinic settings (*n* = 5), in schools, between a combination of clinic and community-based settings, and within university/research settings.

Home visitor models were also popular within the current review (*n* = 25). Typically conducted by public health or nursing professionals, these intervention and prevention initiatives considered engaging service users within their own home as central to the therapeutic model as well as successful outcomes. As the name implies, home-visitation initiatives are typically conducted within the service user’s home; however, there are examples of these programs also engaging with the family in other settings, such as hospitals, prior to the discharge of a mother following childbirth.

Other key mechanisms to note include programs based on cognitive behavioral therapy (CBT) models and pre-school/daycare-based programs. The CBT-based initiatives included in the current review were clinic or home-based services for children who were victims of sexual abuse. Early intervention programs were offered within pre-school/daycare-based programs often for children deemed “at-risk” due to poverty. Pre-school-based programming was also offered in conjunction with programming provided to the service users within their own home or within community-based settings and home.

Several treatment interventions or prevention initiatives and their key mechanisms did not fall under the aforementioned categories of parent education programming, dyadic parent-child relational interventions, or home visitation. These included direct treatment initiatives for mothers with depression and/or other mental disorders. These programs were offered to mothers in mental health clinics [73, 75–77, 119, 120] or via phone-based intervention [71]. Other studies investigated family-based interventions provided in residential settings [53, 116, 121], or focused on wrap-around type services [42], or that targeted increased family access to community resources for parenting [112]. Still other studies examined experiential interventions including music therapy [122], play-based therapy [123], and interventions targeting educators in order to indirectly impact the child’s wellbeing [43, 98, 109].

Our analysis considered the role of fathers as potential contributors to therapeutic interventions. Fathers continue to be only marginally involved in research pertaining to treatment, early intervention, and prevention programs for children (see online Additional file 3). Only a few of the studies included in the current review [65, 124–126] focused on fathers and/or had a high level of participation (i.e., within direct service or completion of measures) from fathers. Some studies examining the role of “caregivers,” “parents,” or “families” within interventions offered a gendered analysis (i.e., differential impact of mothers/fathers on outcomes) of the intervention [44, 68, 69, 74, 101, 106, 119, 127–133], while most studies relied almost exclusively on mothers reporting on assessment and outcome measure and/or participation in treatment interventions [30, 32, 35, 38, 46, 60, 75, 134–138] or mothers represented the vast majority (i.e., 84%, 90%, 94%, 96%) of the parent/caregiver participant from the family in the included studies [48, 84, 88, 114–116, 118, 139–146]. Understanding the differential impact of mothers and fathers within these interventions was further frustrated by studies [34, 36, 122, 147–151] that failed to provide clear indication of the specific composition of the parent/caregiver participants by providing numbers of mothers and fathers.

### Outcome measures

Outcome measures spanned a range of children’s social, emotional, cognitive/intellectual, and behavioral functioning, as well as a range of parental social, emotional, and behavioral functioning. Outcome measures were a combination of parent self-completed measures or reports pertaining to their own functioning, parent-completed measures pertaining to the child’s functioning, parent-completed ratings of the nature/quality of the parent-child interactions or relationship, educator-completed measures or reports pertaining to the child’s functioning, performance-based testing of the child’s functioning by psychologists, as well as third-party observation of parental functioning, child functioning, and/or the nature/quality of the parent-child interactions or relationship. There appeared to be little consensus on the measures used (e.g., a gold standard) even within studies that were similar in nature, making it difficult to interpret the impact of interventions on outcome. Our team looked at the measures for a few of the most popular interventions to understand the landscape of measures employed including parent-child interaction therapy (PCIT), a dyadic-based intervention and incredible years (IY), a parenting group intervention, two modalities representing different intervention mechanisms. In addition to frequency of studies within each intervention, these approaches are manualized and theoretically should have a greater rationale between aim of intervention and outcome measures.

In relation to PCIT, multiple measures were used for child functioning/behavior, parent functioning, and/or parenting and for the parent-child relationship. Across 11 studies that explicitly stated their model of intervention to be PCIT, there was an array of child functioning measures with only two measures showing any consistent use across studies including the ECBI [47, 130, 152–156] and the Child Behavior Checklist [124, 155–157], with a variety of other measures used with little consistency across the studies. Refer to Additional file 3 for a list of the other child-based measures employed within the PCIT studies [124, 130, 146, 153, 154, 157, 158]. Although some of the heterogeneity in child measures may be accounted for by the differing ages of children across the research, this was largely not the case as most studies were providing interventions to children approximately 4 years of age. Furthermore, heterogeneity of measure due to age would not explain the diversity of measure used for parenting and/or parental well-being.

A similar pattern was noted in the diverse usage of parent functioning/parenting measures. The Beck Depression Inventory (BDI) was the most often used outcome measure for parent (i.e., maternal) mental health [124, 130, 153, 158], and the Parenting Stress Index (PSI) was the most often used in the studies for parenting-related factors [146, 152, 156]. Refer to the Additional file 3 for a listing of the other measures employed with the studies [130, 146, 156]. Interestingly, despite PCIT’s central therapeutic mechanism purported to be the parent-child dyad, only three of the 11 PCIT studies employed measures to determine change in this relationship, which included the Dyadic Parent-Child Interaction Coding System-III (DPICS-III) [124, 155] and the Interview Schedule for Social Interaction [157].

A similar pattern of heterogeneity of measures was found when we examined the nature of the 15 studies [34, 36, 38–40, 48, 51, 62, 84, 129, 132, 137, 140, 141, 149] that explicitly stated their model of intervention to be the IY program. In relation to the child’s functioning, the ECBI was the most commonly used [34, 36, 38–40, 51, 138] followed by the Strengths and Difficulties Questionnaire [34, 39, 40, 51]. Refer to Additional file 3 for other child-focused measures used in these studies [34, 38, 40, 51, 84, 129, 132, 137, 140, 141]. The BDI was the measure most often used within the IY studies to gauge parent (typically maternal) mental health and the PSI was the most common outcome measure for parenting-based factors [34, 39, 51, 137], although a variety of other measures were used across studies [34, 38, 39, 51, 62, 129, 137]. Interestingly, measures of the parent-child relationship were employed in IY studies [39, 40, 51, 62, 84, 129, 132, 140] more commonly than in the PCIT, despite these interventions typically being more known for a focus on parent education, skill building, and behavior change in the child. The most common measure was the DPICS-III used in multiple studies [39, 40, 51, 62], although several other measures were used across the studies [62, 84, 140].

## Discussion

The primary strength of this scoping review is its comprehensive nature, including 162 articles following the full-text assessment of 532 studies; the only review of its type ever conducted in relation the 0–5 population. Due to its breadth, we were able to achieve the goals of the scoping review methodology as articulated by Arksey and O’Malley [24]. That is to examine and report on the extent, range, and nature of mental health-related interventions and prevention initiatives for children 0–5 and their families. We comprehensively examined trends in publication dates, geographic locations, institutional settings of the research, research methodologies employed, level of the intervention within a public health framework, key therapeutic mechanisms underpinning the interventions, and the outcome measures utilized.

### Timing of publication rates

Publication dates showed little growth from the mid-1970s throughout the 1980s, then a modest increase occurred in the 1990s followed by an upward spike around 2000 and a major growth spike around 2007. To make sense of these trends, we considered the broader changes occurring within medicine, psychiatry, and the mental health field during this overall 40-year time period. During the mid-1980s and early 1990s, IMH shifted from a field relying on practitioners’ authority-based decision-making to inform treatment decisions toward a decision-making process incorporating empirical evidence, commonly referred to as evidence-based medicine (EBM). The upward trend in publication frequency appears consistent with the growth curve for the increased usage of the term EBM within the medical literature [159]. Consistent with the EBM movement in 1992, the US government established the Substance Abuse and Mental Health Services Administration (SAMHSA) with the objective of making substance use and mental disorder information, services, and research more accessible. The timeframe of the first publication spike occurred between the years 1999 and 2004 coinciding with the Hawaii Department of Health’s launching in 1999 of the Hawaii Empirical Basis to Services Task Force (HEBSTF). This task force provided an interdisciplinary evaluation of interventions common in children’s mental health by using research on controlled treatment studies to produce what is commonly referred to as the *Hawaii Blue Menu* [160]. The next publication spike in the field occurred between 2005 and 2008, coinciding with SAMHSA’s 2007 launch of the National Registry of Evidence-based Programs and Practices (NREPP), an online searchable database to help the public learn more about available evidence-based programs and inform their decision making.

Other factors impacting research on IMH include the launch of the first book titled Infant Psychiatry [161] and the launch of key journals including the *Zero*-*to*-*Three Journal* in 1977 and *Infant Mental Health Journal* in 1980. Rising publication rates in the 2000s may also be partially explained by researchers having deliverables from studies investigating the prevention-based *Head Start* programs of the 1980s and 1990s, most of which were published between 2003 and 2012 [52, 62, 97–99, 101, 102, 107–112]. Furthermore, for attachment-informed interventions, the codification of Ainsworth’s Strange Situation was the impetus for observational and longitudinal studies on the importance of primary caregiver interactions in the early years [2].

### Geographic locations

The majority of the studies were based on samples of children, caregivers, and/or families residing in Western or English-speaking countries including the USA (55%), Australia (17%), the UK (9%), and Canada (4%) representing 85% of the studies. The majority of studies were conducted by research teams comprised of, or led by Western-based researchers (i.e., researchers identifying their affiliations at university settings within Western countries). Studies conducted on Romanian-based populations of infants [162–164] were conducted by a team of US-based researchers, and the studies conducted on South African [93, 94] and Pakistani [80] children and families were led by UK-based researchers. The disproportionate rate of Western publications may arise as an artifact of our studying children’s mental health and more specifically psychosocial interventions for this population, which are both decidedly Westernized concepts [165]. However, such a pronounced imbalance seems inconsistent with infant and maternal mental health’s world-wide popularity, as evidenced by the growing memberships in international organizations with global representation, including the World Health Organization, Pan American Health Organization, World Association for Infant Mental Health, and the International Association for Child and Adolescent Psychiatry and Allied Professions. Our results support the conclusions of other researchers, such as Iverson [166], Maj [167], and Singh [168], who outlined concerns regarding the paucity of research from non-Western countries.

### Research designs

RCTs were the overwhelming methodology of choice for researchers, representing 51% (*n* = 82), or 54% (*n* = 87) with clustered RCTs. When quasi-experimental designs are included, controlled intervention studies represented 77% (*n* = 124) of all studies. This figure is not surprising considering that our review focused on psychosocial interventions by researchers with related research agendas to establish program effectiveness in the relatively new field of IMH. Although US researchers conducted the majority of RCTs (57% of RCTs), this methodology was also employed proportionally in Australia, the UK [34, 36, 38, 40, 49, 103], Canada [117, 134, 139], Romania [162–164], Netherlands [59, 169], Sweden [170, 171], China [150], Germany [161], Ireland [51], Israel [126], Puerto Rico [130], and South Africa [138, 94]. Therefore, it is not the US-based research driving the overrepresentation of RCTs within the field, as this methodology is used proportionally by other countries. In addition to our focus on psychosocial interventions, the preponderance of RCTs and quasi-experimental designs within our review may be due to the EBM climate, organizational pressures to use “evidence-based” programming, and the related financial incentive from funding agencies as different approaches compete for “market share” [172–174].

Numerous gaps in the literature base as it relates to research methodologies were detected. For example, there were a relatively small number of long-term follow-ups on RCTs [7, 75, 87, 90, 91, 135, 155, 175, 176], clustered RCTs [79], and/or quasi-experimental designs [81, 92]. In order to understand the impact of treatments, and in particular prevention-based programs, it is imperative to track the impact of an intervention over time. These methods, along with larger epidemiological studies, would allow researchers to accurately determine what risk factors carry what weight in the onset and continuance of disorders and how those who experienced intervention and/or prevention may fair developmentally, relative to their controls. In addition, important contextual variables, such as gender, age, ethnicity/culture, language and geographic setting, and how these interact with therapeutic mechanisms to influence the desired outcome(s) of intervention, remain unexplored. These questions may require newer methodologies, such as mixed methodologies and qualitative meta-analyses to account for this dynamic interplay between context, therapeutic mechanism, and outcome [177].

### Level of intervention, and populations

Interventions, and/or the populations targeted, aligned in general with the nested public health preventative levels of risks framework [1, 20, 21]. Most research focused on selective prevention (58%; *n* = 94) followed by indicated prevention (26%; *n* = 42) with only a modest commitment to universal programming (7%; *n* = 12) and direct treatment interventions (9%; *n* = 14). Thus, a major finding of the review is that over half of the interventions are in the selective category.

While this aligns with the field’s focus on prevention, the definition of “at-risk” varied widely within and between the selective and indicated categories. There was no consensus on definitions of risk as it pertained to theoretical or probabilistic risk (belonging to a target group at-risk), versus actual risk (subclinical but detectable deviant behavior problems), and in certain studies the populations studied had variations of both selective and indicated risk. In the indicated category, subclinical thresholds for risks as defined by various child and adult measures need to be better defined and agreed upon by researchers as clinically relevant or not. The diversity of risk definitions between intervention studies is a key area for further research in order to achieve greater clarity as to what specific risk factors most accurately predict future mental health issues thereby allowing more effective targeting by selective or indicated interventions. Despite these concerns, there is strong evidence to show that early interventions with children deemed at-risk for the most common presenting concerns of early childhood are the most cost effective [178] and more effective than similar interventions carried out during later periods of development, such as adolescence [179].

Our results showed a paucity of truly universal programs for infants, caregivers, and families regardless of the presence of unique risk factors and/or problems. The appeal of universal programs lies in their ability to reduce access-to-service barriers and to stigma arising from being considered a member of an “at-risk” group and/or being diagnosed with a mental disorder [180]. Higher implementation and evaluation costs, relative to more targeted prevention initiatives and lower effect sizes compared to more direct interventions, [181] are some of the barriers to universal program implementation.

Research pertaining to direct interventions for preschoolers diagnosed with a mental disorder was only marginally more popular than universal programming. Mental health professionals are reluctant to formally diagnose children due to the questionable validity of the *Diagnostic and Statistical Manual* (*DSM*-*V*; *APA*, *2013*) for this age group, prompting the development of the age sensitive-diagnostic classification system, *Diagnostic Classification of Mental Health and Developmental Disorders of Infancy and Early Childhood*, *Revised Edition* DC:0-3R [182] and now DC:0-5 [183] which are starting to be studied for their developmental validity. Currently, the field of children’s mental health has been shifting away from organizing treatments and associated intervention research around psychiatric diagnosis, favoring instead to rely on symptom ratings garnered through the use of standardized measor observations /or observations [160]. We noted that close to 30% of the interventions included children with problem severity levels surpassing the sub-clinical range, but who continued to be described as “aggressive,” “defiant,” or as experiencing “conduct difficulties” rather than being engaged in a formal diagnostic process.

Due to the limited number of studies that sampled populations of children 0–5 with formal diagnoses, it is difficult to understand claims made in relation to popular intervention programs regarding their effectiveness to remediate specific mental disorders. Intervention programs, such as PCIT or IY, often attest to their effectiveness to re-mediate specific mental health categorical disorders (e.g., attention-deficit/hyperactivity disorder [ADHD]) while few studies actually examined populations of children 0–5 years with formal psychiatric diagnoses. In relation to PCIT, these studies include ADHD [47, 130, 154], oppositional defiant disorder (ODD) [152, 153, 155], or major depression [146, 158]. In relation to incredible years (IY), these studies include ADHD or conduct disorder [48], or ODD [137]. The vast majority of children involved in these studies were noted as displaying elevated behavioral difficulties via standardized measures such as the ECBI or displaying behavioral problems, aggressive, or having conduct difficulties without these terms being adequately operationalized. As developmentally sensitive tools such as the DC: 0-5 and the Preschool Age Psychiatric Assessment (PAPA) [14] are being developed, our findings have identified a gap in research regarding how researchers might achieve greater rigor and consistency in how they operationalize child problem categories.

### Key therapeutic mechanisms and outcomes

The results of our review provided a picture that parent education/skills groups, dyadic parent-child relational interventions, and home visitation programs represent the three main pillars of direct practice for children 0–5 at risk for, or experiencing mental disorders. Each approach is distinct in its therapeutic mechanism having a discrete ideological/theoretical foundation, but interestingly, all three seek similar aims for children, parents, and families. A fourth pillar, the preschool/daycare-based initiatives, was distinct in mechanism, relative to these direct practices, by being upstream targeting infants and families “at-risk” representing the bulk of the evidence-based early intervention and/or prevention initiatives. The fifth pillar, CBT approaches, is relatively new but represents parentally mediated skill-based practices for anxiety and trauma, indirectly tapping into the child-parent relationship.

Each major result category could have been cross-referenced within and between other categories, but these sub-analyses were beyond the scope of this paper. For example, dyadic therapies made up 32 and 24% of the indicated and selective categories respectively, suggesting that some researchers may have perceived this modality as applicable to theoretical versus actual risk in the child, or the parent or the parent-child relationship. Setting may influence choice of modality, but it is not clear if this is driven by consumer or researcher preferences as well as recruitment portals. A key therapeutic ingredient lacking in IMH research and intervention was paternal involvement. Barriers to fathers’ participation may be associated with the incompatibility of the schedules maintained by researchers and mental health professionals, underestimation of impact of fathers’ roles in attachment theory, and stereotypes about male roles [184]. Finally, the choice of outcome measures employed by researchers showed great heterogeneity even within similar interventions at times depending on the perceived therapeutic mechanism.

### Limitations

There were several limitations to our study that should be highlighted. Firstly, despite the ambitious breadth of this scoping review, it is not meant to be exhaustive in nature. It was intentionally decided that due to our exclusion/inclusion criteria, some interventions with their associated populations were left out. We excluded young children with severe developmental challenges such as children primarily diagnosed with autism spectrum disorder or fetal alcohol syndrome, as these children and families required interventions of a qualitatively different nature than children with socioemotional difficulties. It may be argued that they should have been included in the direct treatment category as many of these children have important socioemotional comorbidities. Secondly, due to the descriptive nature of scoping reviews, we were unable to provide further mechanisms beyond general trends, thus putting together interventions that have vastly different key therapeutic mechanisms. For example, in the field of parent-child interactions, the dyadic child-parent psychotherapies have a different focus than dyadic parent-child interactional therapy. Thirdly, there were many invaluable cohort studies informing the processes of child development and the role of risk and protective factors but due to the study criteria of only including intervention studies, no cohort studies were included in this review.

Lastly, a key limitation of the current scoping review is a challenge common to review studies. The length of time that elapsed between the end-date of the search (i.e., December 31, 2012) and the time of publication, spanning a 7-year time frame, limits our ability to fully report on the true landscape of the research pertaining to interventions for at-risk young children (0–5). Establishing review criteria with such a broad scope to reporting on all intervention studies for this population create a unique challenge of identify a tremendous amount of research, requiring our research team to balance the need for rigorous analysis and reporting with expedience in publication, as new research, not captured in our search parameters, is ever emerging within this burgeoning field, as has been the trend since the early 2000s (see Fig. 2). In order to determine if this trend continues, the research team extended our hand search of key journals including *Infant Mental Health Journal*, *Child Development*, the *Journal of the American Academy of Child and Adolescent Psychiatry*, and the *Journal of Child Psychology and Psychiatry* between January 12,013 and February 28, 2019. Our hand search confirmed the upward trend continues for intervention research to be conducted with this vulnerable population by identify 39 new studies [185–223]. This finding underlines the importance of conducting future and ongoing reviews that adhere to best practices in scoping and/or systematic review, the need for which cannot be diminish by our hand search. Although, we did not integrate those articles identified through the hand search into the main analysis of the current scoping review, we did include the findings within the full table that includes the results of the 162 studies (see Additional file 3). While none of the original interventions in the 162 studies were delivered in an online or digital format, some of the intervention studies since 2013 were delivered online (216,237). Our intention here is only to locate this recent research within the broader landscape of research, but we underline caution in drawing any conclusions related to the field of research between 2013 and 2019 based on 39 drawn from only four specialized journals.

## Conclusions

Through this review, we were able identify key gaps in the early years mental health intervention literature, including the need for future research from non-Western countries, better definitions of risk factors and associated outcomes, and the role of fathers’ involvement in IMH initiatives. This scoping review was able to examine and describe the intervention literature within the parameters of an accepted model of public health [1, 20, 21] with an overlapping “nested” view of prevention and intervention. As such, both the current scoping review and the public health framework chosen are heuristic concepts which reflect the dual realities of prevention and intervention continua in the early years, but the model is more descriptive rather than predictive. More research on risk factors, therapeutic mechanisms, and outcomes is needed to separate out children and families with differing trajectories or “developmental assets” in order to match need to risk level and build more comprehensive “ecologically valid” intervention models.

## Additional files

Additional file 1: PRISMA-ScR Checklist. (DOCX 83 kb)

Additional file 2: MEDLINE search strategy. (DOCX 13 kb)

Additional file 3: Overview of studies included in review. (DOCX 143 kb)

## Abbreviation

IMH: Infant mental health
