# Supplementary material for: A scoping review of mental health prevention and intervention initiatives for infants and preschoolers at risk for socio-emotional difficulties
Source: Syst Rev. 2019 Jul 23;8:183. doi: 10.1186/s13643-019-1043-3 (PMC6651971; doi:10.1186/s13643-019-1043-3)
Supplement: Supplementary file 2 — MEDLINE search strategy. (DOCX 13 kb) [file 13643_2019_1043_MOESM2_ESM.docx]

**Additional file 2.** *MEDLINE search strategy*

| 1. social adjustment/ |
| --- |
| 2. anxiety disorders/ |
| 3. exp anxiety disorders/ |
| 4. exp mental disorders diagnosed in childhood/ |
| 5. mood disorders/ |
| 6. exp depressive disorder/ |
| 7. aggression/ |
| 8. mentally ill persons/ |
| 9. anxiety.tw. |
| 10. (internali* adj3 (symptom* or disorder* or difficult* or behavio?r* or problem*)).tw. |
| 11. (externali* adj3 (symptom* or disorder* or difficult* or behavio?r* or problem*)).tw. |
| 12. (internali* adj3 syndrome).tw. |
| 13. (externali* adj3 syndrome).tw. |
| 14. (aggressi* adj5 (symptom* or disorder* or difficult* or behavio?r* or problem*)).tw. |
| 15. disruptive behavio?r*.tw. |
| 16. hyperactiv*.tw. |
| 17. ((anti-social or antisocial) adj3 (symptom* or disorder* or difficult* or behavio?r* or problem*)).tw. |
| 18. (oppositional adj3 (defian* or disorder*)).tw. |
| 19. (emotional adj2 behavio?r* disorder*).tw. |
| 20. social behavio?r* disorder*.tw. |
| 21. (?attachment adj3 disorder*).tw. |
| 22. or/1-21 |
| 23. infan*.tw. |
| 24. preschool.tw. |
| 25. babies.tw. |
| 26. baby.tw. |
| 27. neonat*.tw. |
| 28. child, preschool/ |
| 29. exp infant/ |
| 30. or/23-29 |
| 31. (multidisciplin* adj3 (program* or intervention* or educat* or therap*)).tw. |
| 32. (interdisciplin* adj3 (program* or intervention* or educat* or therap*)).tw. |
| 33. (collaborat* adj3 (program* or practice* or intervention* or educat* or approach* or therap*)).tw. |
| 34. (behavio?r* adj (therap* or intervention* or train* or educat* or program*)).tw. |
| 35. (cogniti* adj3 therap*).tw. |
| 36. (parent* adj2 (therap* or intervention* or program* or train* or educat*)).tw. |
| 37. (famil* adj2 (therap* or intervention* or program* or train* or educat*)).tw. |
| 38. (attachment adj2 (therap* or intervention* or program* or train* or educat*)).tw. |
| 39. cbt.tw. |
| 40. parent-child relations/ |
| 41. mother-child relations/ |
| 42. father-child relations/ |
| 43. "Early Intervention (Education)"/ |
| 44. behavior therapy/ |
| 45. cognitive therapy/ |
| 46. child guidance/ |
| 47. or/31-46 |
| 48. 22 and 30 |
| 49. 47 and 48 |
