# Supplementary material for: A scoping review of mental health prevention and intervention initiatives for infants and preschoolers at risk for socio-emotional difficulties
Source: Syst Rev. 2019 Jul 23;8:183. doi: 10.1186/s13643-019-1043-3 (PMC6651971; doi:10.1186/s13643-019-1043-3)
Supplement: Supplementary file 3 — Overview of studies included in review. (DOCX 143 kb) [file 13643_2019_1043_MOESM3_ESM.docx]

**Online Appendix** Overview of studies included in review

| **Author and year of publication** | **Study Design** | **Study Location /Primary Setting** | **Description of study population** | **Mean Age** | **n** | | **Name and Aim of Intervention** | **Outcome Methods/Measures** |
| --- | --- | --- | --- | --- | --- | --- | --- | --- |
| Abikoff et al. (2015) [ref 185] | RCT | USA/ School | Preschoolers with ADHD. | Children: 3.0 to 4.11 years. | 164 children | | New Forest Parenting Package (NFPP): fosters constructive parenting targeting ˅ ADHD-related dysfunctions in attention and impulse control | Teacher and parent ADHD ratings on Conners scales, clinician ADHD ratings using ADHD-Rating Scale-IV, preschool version of NYTRS, Delay of Gratification-Cookies Delay Task, PPI, GIPCI-R, PSI-R, Parent Perceptions of Parent Efficacy Scale, CSQ. |
| Abrahamse et al. (2012)  [ref 47] | Quasi-exp repeat measures | Netherlands / Clinic | Mothers & children with externalizing beh problems. | Children: M=4.7, 5.2 (I,C) years;  Mother: M=34.9, 36.3 (I, C) years. | 37 mother--child dyads. | | **Parent-child interaction Therapy (PCIT):** dyadic intervention targeting ˅ child beh problems. | Eyberg Child Behavior Inventory (ECBI). |
| Altmaier & Maloney (2007)  [ref 147] | Quasi-exp pre-post | USA / Clinic & community-based | Single parents of Caucasian ethnicity getting divorced and their children. | Children: M=47 months; Parent: M=33.58 years. | 12 parent-child dyads. (no gender breakdown given for parents) | | **Mindful parenting:** 15 hours of treatment provided over 12 weeks targeting ˄ parent mindfulness. | Toronto Mindfulness Scale. |
| Ansari et al. (2017)  [ref 186] | Quasi-exp pre-post | USA/ School & community-based | Low-income Latino children who experienced public school pre-K or child care via subsidies (center-based care) at age 4. | Children: measured at age 4 and in third grade. | 11902 children | | Publicly funded preschool programs: ^ school-readiness of young Latino children in poverty | Pre-school entry and end of preschool: LAP-D, DECA, Kindergarten entry: OLPS–R, Third grade: GPA, Math test score, Reading test score by Florida Comprehensive Assessment Test. |
| Armstrong et al. (1999)  [ref 56] | Quasi-exp repeat measures | Australia/ Home | Mothers & children exposed to risk factors (e.g., domestic violence, single parenthood, parental MH, low SES). Significant group of Aboriginal ethnicity. | Children: Described only as ‘newborns’. | 174 mothers | | 6 month intervention facilitated by nurses targeting ˅ maternal PDD, ^ parent-child interaction, ^ parent child care knowledge, and ^ quality of home environment. | Parent reports on child's beh and survey of parenting changes. |
| Asscher, Hermans, & Delovic, (2008)  [ref 97] | Quasi-exp | Netherlands / Home | Dutch mothers with children under 5 years. No requirement for psychosocial risk factors. | Children: 30.1, 30.6 (I, C) months; Mother 31, 34 (I, C) years. | 54 mothers | **Home Start:** home visiting program targeting ˅ child beh problems, ^ maternal well-being, ^ perceived parental competence, ^ parenting consistency, and ^ maternal sensitivity. | | Parenting Stress Index-Revised (PSI), Nijmegen Parenting Questionnaire (NCSQ), Parenting Dimensions Inventory (PDI), Child Behavior Checklist (CBCL), Erickson Standardized Rating Scales for Home Observation, Coder Impressions. Inventory |
| Bagner et al. (2013)  [ref 124] | One group pre-post with f/u | USA/ Home | Hispanic mothers & children at-risk for beh problems. | Children: M=13.33 months;  Mother: M=31.43 years. | 7 parent-child dyads | | **PCIT:** dyadic intervention targeting ˅ child beh problems. | Dyadic parent-child Interaction coding system-III (DPICS-III), Brief Infant-Toddler Social and Emotional Assessment (BITSEA), and CBCL. |
| Bagner & Eyberg (2003)  [ref 152] | Quasi-exp pre-post with f/u | USA/ Clinic | Parents of children Dx ODD. Involved vs absentee fathers. Ethnic minorities in both groups. | Children: M=4.45, 4.41 (I, C) years. | 107 families | | **PCIT:** dyadic intervention targeting ˅ child beh problems and ˅ parenting stress. | ECBI, PSI, Beck Depression Inventory (BDI). |
| Bain (2014) [ref 187] | RCT | South Africa/ Community-based | High-risk mother–infant dyads. | Infants: Experimental Group 1: M= 22.2 months, Experimental Group 2: 20.7 months, Experimental Group 3: M= 2.1 months, Control Group: M= 20 months. | 22 mother-infant dyads | | New Beginnings: dyadic intervention targeting ^ mothers’ ability to attune to her infant’s needs,  ^ mother’s ability to acknowledge her infant’s separateness, ^ mother’s  range of contingent responses to her infant, ^ mother’s capacity for mentalization. | Reflective Function Scale (RF) as scored on the PDI, mother’s depression and anxiety by Kessler-10, Emotional Availability Scales (EA), infant’s development by Griffiths Scales. |
| Baker (2018)  [ref 188] | Path analysis | USA/ Community-based | Nationally representative sample of Head Start children from the Family and Child Experiences Survey of 2009 (FACES). | Children: 53.31 months. | 3349 children | | Head Start: ^ mother warmth and shared book reading, ^ children’s EF, and ˅ behavior problems. | Pencil-tapping task, BPI, abbreviated version of Center for Epidemiological Studies of Depression Scale, Child Rearing Practices Report, Family Economic Risk Index. |
| Baker et al. (2015)  [ref 189] | RCT | USA/ Tele-Intervention requiring internet access | 15 adoptive dyads with children experiencing developmental/intellectual, emotional, behavioral, and/or attachment-related challenges, most having experienced in utero maltreatment. | Children: M= 42 months Parents: M= 39 years. | 15 adoptive dyads | | Online Emotional Attachment and Emotional Availability (EA2): postadoption support, ^ parent-child emotional attachment and emotional availability, ˅ child behavior problems, ^ attachment security | Emotional Availability Scales, Fourth Edition (EA Scales, Emotional Availability-Self-Report (EA-SR), Emotional Attachment & Emotional Availability Clinical Screener (EA2-CS), The Attachment Q-Sort, Version 3.0 (AQS), Parenting Stress Index (PSI), Child Behavior Checklist-Parent Report for ages 1.5 to 5 years (CBCL). |
| Barkley et al. (2000)  [ref 127] | Quasi-exp pre-post (4 group design | USA/ School | Low SES parents of preschoolers with disruptive aggressive beh. | Children: M=4.9 years; Mother: M=28.2 years; Father: M=31.8 years. | 157 children | | Parenting group and treatment classroom targeting ˅ child beh problems, ^ parent competency and ˅ parenting stress. | CBCL & Conners’ Teacher Report Form (TRF), Home Situations Questionnaire (HSQ), Normative Adaptive Behaviour Checklist (NABC), Parenting Sense of Competence Scale (PSOC), PSI, Parenting Practices Inventory (PPI), School Situations Questionnaire (SSQ), Self-Control Rating Scale (SCRS), Social Skills Rating Scale (SSRS), observation of mother-child interaction during free play task, and classroom beh observations. |
| Barnet et al. (1996)  [ref 57] | Quasi-exp pre-post with f/u | USA / Clinic | Pregnant teen mothers who are experience MH issues. Most were low SES single parents of African American ethnicity residing with their mothers. | Children: Intervention began during prenatal period. Mothers: Range 12-18 years. | 125 mothers | | Parenting skill intervention targeting ˅ depression, ˄ perceived social support and ˅ perceived stress. | Not specified. |
| Baydar et al. (2003)  [ref 62] | Quasi-exp pre-post | USA/ School | Ethnically diverse parents with MH issues whose child enrolled in Head Start program. | Children: M=56.2 months. | 882 mothers | | **Incredible Years (IY):** parenting group targeting ˅ child academic, social, emotional & beh problems and ^ parenting skills. | PPI, DPICS-Revised, Coder Impression Inventory  (CII) and independent observation of parent-child interaction. |
| Bayer et al. (2007)  [ref 180] | Clustered RCT | Australia/ Clinic | Diverse SES group of parents of preschoolers | Children: M=7 months; Mother: M=33.2 years. | 733 mothers | | **Toddlers without Tears**: parent group & ‘well baby’ visits targeting prevention of preschool beh problems. | CBCL, Parent Behavior Checklist (PBC), Depression Anxiety Stress Scales (DASS). |
| Beardslee et al. (2010)  [ref 82] | Mixed methods design | USA / Community-based | Head Start Staff, (Social Workers and Early Childhood Educators). | Not indicated. | Unclear Sample size drawn from surveys, interviews & focus groups. | | **Family Connections Intervention:** a psychoeducational program for Head Start staff targeting ˄ capacity for staff to deal with depression in children and families. | Focus groups with program staff on interventions benefits. Third party observation within classroom using the Early Childhood Environmental Rating Scale Revised (ECERS-R) and Caregiving Interaction Scale (CIS). |
| Beeber et al. (2004)  [ref 52] | RCT | USA/ Home | Low SES mothers with depressive symptoms. | Children: Range 1-5 years; Mother: M=26.6 years. | 16 mothers | | **Early Head Start:** 8 sessions by nurses at home & 8 phone sessions targeting ˅ maternal depression. | Centre for Epidemiologic Studies Depression Scale-Revised (CESD-R), and observation of maternal-child interactions through unstructured play coded by researcher. |
| Beeber, Perreira, & Schwartz (2008)  [ref 63] | Quasi-exp pre-post | USA / Home | Low income mothers with depressive symptoms | Mother: M=27.47 years. | 57 mothers | **Nurse Home Visiting** targeting ˅ depressive symptoms. | | CES-D and observation of mother-child interactions. |
| Bernard et al. (2012)  [ref 114] | RCT | USA/ Home | Parents & maltreated, child welfare referred children. Most were African American, mothers. | Children: M=19.1 months; Parents: M=28.4 years. | 120 children; 113 parents  (98% were mothers). | | **Attachment and Biobehavioural Catchup (ABC):** manualized session for parents & children targeting ^ attachment quality. | Strange Situation Classification (SSC). |
| Bernard, Simons, & Dozier (2015)  [ref 190] | RCT | USA/ Home | Mothers involved with CPS following allegations of neglect and low-risk mothers with no history of CPS involvement. | Mothers: 31.6 years. | 86 mothers | | Attachment and Biobehavioral Catch-up (ABC ): 10-sessions to ^ maternal sensitivity among CPS referred mothers. | Event-related potential (ERP): data recorded by continuous EEG, Qualitative Scales of the Observational Ratings of the Caregiving Environment (ORCE), Depression subscale of the Brief Symptoms Inventory. |
| Bierman et al. (2008)  [ref 98] | RCT | USA / School | Educators of preschoolers | Children: M=4 years. | 356 children | | Training materials provided over a 1 year period to educators targeting ^ ability of educators to use Head-start materials. | Measures not specified related to the methods of child assessments, teacher ratings, parent ratings, and direct observations. |
| Bierman et al. (2014)  [ref 191] | RCT | USA/ Community-based | Children who experienced Head Start enriched with the REDI evidence-based curricula and teacher support and children who received “usual practice” Head Start. | Children: M=4.59 years. | 356 children | | Research-based, Developmentally Informed (REDI): ^ language and emergent literacy skills, ^ development of social-emotional skills | Multimethod, multi-informant assessment battery assessing language and emergent literacy skills, learning engagement, social competence, aggressive behavior, and kindergarten context. |
| Bierman et al. (2015)  [ref 192] | RCT | USA/ Home | Families from Head Start centers. | Children: M= 4.45 years. | 200 children and parents | | Research-based Developmentally Informed (REDI) Parent program (REDI-P): ^ sustained effects of REDI by partnering with parents to provide learning support at home that parallels and reinforces REDI classroom programming over transition to kindergarten. | Four measures to assess child language and emergent literacy skills, three teacher-rated measures to assess social emotional adjustment, parent support for learning assessed by parent report, videotaped observations of parent–child interactions, and observations conducted in the home. |
| Black et al. (2007)  [ref 175] | Long-term f/u to RCT | USA/ Home | Low SES parents & children. African-American ethnicity. | Children: M=37.6 months; Mothers M=27 years. | 130 children | | Intervention targeting ˅ child beh problems, ^ positive child developmental outcomes and ^ child school readiness. | CBCL, TRF, Wechsler Intelligence Scale for Children (WISC), Wide Range Achievement Test-Revised (WRAT) and Nurse examination of child at 8 years compared to developmental norms. |
| Bor, Sanders, & Markie-Dadds (2002)  [ref 32] | Quasi-exp (3 groups) pre-post with f/u | Australia/ Clinic | Lower SES parents of preschoolers with disruptive beh & ADHD related issues | Children: M=40.41, 39.86, 42.81 months; Mothers: M=28.41, 30.21, 29.72 years; Fathers 31.54, 33.65, 33.03. | 87 families | | Parenting group targeting ˅ child beh problems and ^ parenting skills. | CBCL and TRF. |
| Bosquet & Egeland (2001)  [ref 83] | RCT | USA/ Home & Clinic | Low SES pregnant mothers with depression of diverse ethnicity with most being single parents. | Children: described as infants with no specific age give; Mothers: M=20.51 years. | 110 parent-child dyads | | **Systematic Training in Effective and Enjoyable Parenting (STEEP**): Structured intervention beginning at the clinic with parent education in 2^nd^ trimester followed by home intervention for mother-infant dyad in the home targeting ^ maternal sensitivity to child cues. | CES-D. Videotaped and coded mother-child observation. |
| Bradley et al. (2011)  [ref 99] | RCT | USA / School | Children from lower SES communities with parents with low education and employment levels. Diverse ethnicity including African, American, Hispanic, Caucasian. | Children: Range 3-5 years. | 3001 children | | **Head Start:** based intervention implemented over 3 years targeting ˄ school readiness through ˄ social beh, ^ language and ^ cognition. | Woodcock-Johnson-III Test of Achievement (WJ III ACH), Skills and Positive Approaches to Learning Scale, Bayley Scales for Infant and Toddler Development (Bayley-III) and in-home videotaped and coded child-parent interactions. |
| Bradley et al. (2003)  [ref 139] | RCT | Canada/ Community-based | Parents of children with beh problems of middle SES | Children: M=3.76, 3.84 (I, C) months; Parent in treatment: M= 35.20, 35.88 (I, C) years; Other Parent: M= 37.76, 37.57 (I, C) years. | 222 parents  (84% were mothers). | | **1,2,3 Magic:** parenting group targeting ^ effective discipline and  ˅ parent-child conflict | Leiter International Performance Scale (Leiter-3) in-home videotaped and coded child-parent interactions. |
| Brotman et al. (2003)  [ref 84] | RCT | USA/ Clinic & Home | Urban, ethnically diverse, low SES parents of preschoolers at risk for conduct disorder. Family member with a criminal record. | Children: Range 2.5-5 years. | 30 children and their parents (90% were mothers). | | **IY:** Co-occurring parent & child groups with in-home component targeting ˅ child beh problems and ^ parenting responsiveness. | Videotaped observation of parenting practices coded with Global Impressions of  Parent–Child Interactions (CIPCI-R). |
| Brotman et al. (2005)  [ref 140] | RCT | USA/ Home & Community-based | Ethnically diverse low SES parents of preschoolers at high risk of conduct problems. Most parents were mothers. | Children: Range 33- 63 months; Parents M=36.3 years. | 99 children with their parents (83% were mothers) | | **IY** (Basic) & **Dinosaur Curriculum.** Concurrent parent and child groups with in-home component targeting ˅ child beh problems and ^ parenting capacity. | PPI, The Home Observation  for the Measurement of the Environment (HOME),  Observed Peer Play in Unfamiliar Settings (OPPUS). |
| Brotman et al. (2008)  [ref 141] | RCT | USA/  Home & Community-based | Ethnically diverse low SES parents of preschoolers at high risk for antisocial beh | Children: M=47.52 months; Parent: M=36.3 years. | 92 families (83% of parent participants were mothers) | | **IY** (Basic) & **Dinosaur Curriculum.** Concurrent parent and child groups with in-home component targeting ˅ child beh problems and ^ parenting capacity. | New York Parent Rating Scale-Preschool-Aged (NYPRS-P) and observation of semi-structured, parent–child interactions. |
| Buhler et al. (2011)  [ref 53] | One group pre-post with f/u | Germany / Residential setting | Mothers with  depression and their children. Significant number of mothers (1/3) were single. | Children: M=5.3 years; Mothers: M=35.8 years. | 2379 mothers | | **EFFEKT Parenting Program (formerly the Örebro Prevention Program):** parenting program offered over 3 weeks at mother-child residential health resort with follow-up programming targeting ˅ impact of maternal depression on mother-child relationship. | Social Behavior Questionnaire (SBQ), PSOC, Alabama Parenting Questionnaire (APQ) and PSI. |
| Butz et al. (2001)  [ref 64] | RCT | USA/ Home | Mothers & children exposed in utero to drugs. Most of African- American ethnicity, single mothers. Significant number of kinship caregivers. | Children: M=59 months; Mothers: 28.5 years. | 100 mothers and their children. | | 16 sessions for parents facilitated by nurses targeting ˅ child beh problems. | CBCL, PSI, and research staff examined children for growth and development rates. |
| Bywater et al. (2009)  [ref 34] | RCT | UK/ Clinic | Children at risk of developing conduct disorder in Northern Wales and their parents; families described as disadvantaged | Children: Range 36-59 months. | 104 parents (no gender breakdown given) | | **IY** (Basic): parenting group targeting ˅ child beh problems, ^ parent responsiveness and ˅ parent depression. | ECBI, SDQ, Connors Abbreviated Parent Rating Scale (CAPRS), SCRS, PSI-Short form (PSI–SF), Arnold’s Parenting Scale (PS), BDI, and home observation. |
| Caldera et al. (2007)  [ref 113] | RCT | USA/ Home | Low SES mothers, mostly Native Americans. | Mothers: M=23.4, 23.7 (I, C) months. | 325 mothers and their children. | | **Healthy Families Alaska**: paraprofessional intervention from birth-2 years targeting ˅ child beh problems and ^ overall child health. | CBCL, Bayley-III, The Nursing Child Assessment Satellite Training Teaching Parent-child Interaction Teaching Scale (NCAST), and Conflict Tactics Scale (CTS). |
| Campbell FA, Ramey CT. Effects of early intervention on intellectual and academic achievement: a follow-up study of children from low-income  families. Child Dev. 1994;65(2):684–98. https://doi.org/10.1111/j.1467-8624.1994.tb00777.x. | RCT | USA/  Preschool | Infants born to economically disadvantaged families | Children: M=4.4 months | 111 children | | **Abecedarian Preschool:**  children attended preschool program designed to enhance cognitive, language, perceptual-motor, and social developments | WISC-R, Woodcock-Johnson Pscyho-Educational Battery II, School Records (School Archival Records Systems) |
| Cappleman et al. (1982)  [ref 85] | RCT | USA/ Home | Low SES unmarried teen mothers & their infants of African American ethnicity. | Children: Followed from birth-2.5 years although no specific age data given; Mother: M=15.84 years. | 37 parent-child dyads. | | **Carolina Infant Curriculum:** monthly parent-infant sessions targeting ^ child developmental outcomes, ^ parenting skills, ^ maternal responsiveness and ^ parent-child relationship. | The Denver Developmental Screening Test (DDST), Bayley-III, Stanford-Binet Intelligence Scales (SB) and observation of mother-child interactions. |
| Caramlau et al. (2011)  [ref 71] | One group pre-post | UK / Telephone-based | Mothers with PPD and their children. | No specific age data provided. | 30 mothers | | **Mums 4 Mums:** structured telephone peer-based intervention for mothers experiencing PPD targeting ˅ symptoms of prenatal depression, ˄ coping beh in mothers, ˄ mother-child interaction, ˄ sense of parenting competency and ˄ self-efficacy. | Edinburgh Postnatal Depression Scale (EPDS), Hospital Anxiety and Depression Scale (HADS), PSOC, Dyadic Adjustment Scale (DAS), Emotional Support Questionnaire and Self-Efficacy and the Care Index. |
| Cassibba et al. (2015) [ref 193] | Quasi-exp pre-post | Italy/ Home | Healthy children without any medical complication at birth and their postpartum mothers. | Mother: M= 33.03 years. | 32 mother-child dyads | | Video-Feedback Intervention to Promote Positive Parenting With Discussions on the Representational Level (VIPP-R): ^ mother’s sensitive behavior, and promote a reorganization of mother’s mental representations with respect to attachment. | Adult Attachment Interview (AAI), Emotional Availability Scale (EAS), Strange Situation Procedure (SSP), Video-Feedback Intervention to Promote Positive Parenting with Discussions on the Representational Level (VIPP-R). |
| Cheng S, Kondo N, Aoki Y, Kitamura Y, Takeda Y, Yamagata Z. The effectiveness of early intervention and the factors related to child  behavioural problems at age 2: a randomized controlled trial. Early Hum Dev. 2007;83(10):683–91. https://doi.org/10.1016/j.earlhumdev.2007.01.008. | RCT | Japan/ Home | Low-mid SES mothers & infant dyads of Japanese ethnicity. Most from two parent headed families. | Children: Range 4.5 months-2 years; Mother: M=30 years. | 85 parent-dyads | | Monthly parent & child intervention targeting ˅ child problem beh, ^ parenting skills and ^ parental sensitivity to child cues. | CBCL, observation of mother–child free play. |
| Cho et al. (2013) [ref 194] | Quasi-exp pre-post | Japan/ Home | Preterm mother-infant dyads. | Mother: JIMHP group: M= 32.91 years, Control group: M= 34.50 years. | 41 mother-infant dyads | | Japanese Infant Mental Health Program (JIMHP): promote maternal mental health, mother-infant interaction, social support to mother, ˅ stress relevant to maternal parenting, and child development. | Japanese version of the Center for Epidemiologic Studies Depression Scale (CES-D), Nursing Child Assessment Feeding Scale (NCAFS), modified Social Support Scale, Japanese version of the Parenting Stress Index (JPSI), KSPD, self-report subjective perception of the program. |
| Clark et al. (2008)  [ref 72] | Quasi-exp pre-post | USA / Clinic | Low-mid SES mothers with depression and their children. | Children: M=7.86 months; Mother: M= 28.06 years. | 32 parent-child dyads | | 12 week mother-infant therapy group targeting ˅ depressive symptoms, ˅ parenting stress, ^ quality of the mother-child interaction and ˄ infant developmental outcomes. | Bayley-III, BDI, PSI, and video recorded observed mother-child interactions using the Parent-child Early Relationship Assessment (PCERA). |
| Cohen JA, Mannarino AP. A treatment outcome study for sexually abused  preschool children: initial findings. J Am Acad Child Adolesc Psychiatry.  1996;35(1):42–50. https://doi.org/10.1097/00004583-199601000-00011. | RCT | USA / Clinic | Sexually abused preschoolers | Child age: M=5 years, 9 months. | 43 children | **Manualized CBT-SAP (Sexually abused preschoolers)** targeting ˅ child aggression, ˅ child sadness and ˅ child regressive beh. | | CBCL, Child Sexual Behavior Inventory (CSBI), Preschool Symptom Self Report (PRESS). |
| Cohen JA, Mannarino AP. Factors that mediate treatment outcome of  sexually abused preschool children: six- and 12-month follow-up. J Am  Acad Child Adolesc Psychiatry. 1998;37(1):44–51. https://doi.org/10.1097/00004583-199801000-00016. | RCT | USA / Clinic | Sexually abused preschoolers | Child age: M=5 years, 9 months. | 43 children | **Manualized CBT-SAP (Sexually abused preschoolers)** targeting ˅ child disruptive beh, ˅ sexualized beh and ^ emotional competence. | | CBCL, CSBI, and PRESS. |
| Cohen JA, Mannarino AP. A treatment study for sexually abused preschool children: outcome during a one-year follow-up. J Am Acad Child Adolesc Psychiatry. 1997;36(9):1228–35. https://doi.org/10.1097/00004583-199709000-00015. | RCT | USA / Clinic | Sexually abused preschoolers | Child age: M= 4.68 years. | 67 children | **Manualized CBT-SAP (Sexually abused preschoolers)** targeting ˅ child aggression, ˅ child sadness and ˅ child regressive beh. | | CBCL, CSBI and PRESS. |
| Connell et al. (2008)  [ref 46] | Latent Transition Analysis of RCT Data | USA/ Clinic | Low SES mothers of diverse ethnicity & children at risk for beh problems. | Children: M=29.9 months. | 731 parent-child dyads | | **Family Check-up:** 3 session based on motivational interviewing targeting ˅ child beh problems, ˅ maternal depression and ˅ parent-child conflict. | CBCL and home observation of child beh. |
| Cooper et al. (2002)  [ref 93] | Quasi-exp pre-post | South Africa/ Home | Mothers and their children residing in a very low SES overcrowded settlement of temporary shelters in Cape Town with limited infrastructure and high community risk factors (e.g., violence, subsistence living, illiteracy, transience). | Mother: M=26.1 years. | 32 parent-child dyads. | | **Adapted Health Visitor Prevention Intervention Program:** 6 month intervention provided by para-professionals targeting ^ maternal mood and ^ parent-child relationship. | Structured Clinical Interview of DSM-IV (SCID)-Depression section, observation of mother-child interaction during free play and infants weighed and measured. |
| Cooper et al. (2009)  [ref 94] | RCT | South Africa/ Home | Low SES expecting or new mothers and their children. Most were in cohabiting relationships. | Mother: M=26.1 years. | 449 mothers | | Paraprofessional intervention with mother-child dyads from late pregnancy to child turning 6-months targeting ^ positive mother-infant interactions. | SSC and observations rated with the Parent/Caregiver Involvement Scale (PCIS) by a trained rater. |
| Cooper & Lanza (2014)  [ref 195] | Latent class analysis of RCT data | USA/ Community-based | Children from the age 3 cohort assigned to receive HS. | Children: M= 3.21 years. | 2,449 children | | Head Start: Preschool program for disadvantaged children. | Baseline Characteristics for Defining Latent Class Moderator, Cognitive Outcome Measures, Social-Emotional Competence and Behavioral Outcomes. |
| Cunningham et al. (1995)  [ref 134] | RCT | Canada / Community-based | Families of preschoolers at risk for disruptive disorders; Balance of 2 parent & single parents | Children: Children reported as prekindergarten but no specific age data given. | 150 families | | **Coping Modeling Problem Solving:** parenting group using video vignettes targeting ˅ child beh problems. | CBCL, PSOC and Videotaped and coded mother-child interactions. Parent problem solving measured with non-standardized child beh problem vignette. |
| Dadds & Roth (2008)  [ref 181] | Quasi-exp pre-post with f/u | Australia/ Clinic | Working class/middle SES parents. | Children: Range 3-6 years. | 720 children | | **Reach for Resilience:** parenting intervention targeting prevention of child anxiety. | Teacher completed Social Competence and Behavior Evaluation-Short Form (SCBE), teacher completed Preschool Behavior Play Scale (PPBS), Colorado Childhood Temperament Inventory (CCTI). |
| Dishion et al. (2008)  [ref 35] | Latent growth analysis of RCT data | USA/ Clinic | Low SES mothers of diverse ethnicity & children at risk for beh problems. | Children: M=28.2 months. | 731 parent-child dyads | | **Family Check-up:** 3 sessions based on motivational interviewing targeting ˅ child beh problems, ˅ maternal depression and ˅ parent-child conflict. | CBCL, ECBI, CES-D and Videotaped and coded mother-child interactions. |
| Duggan et al. (2004)  [ref 100] | RCT | USA/ Home | Low SES mothers with depressive symptoms & newborns at risk for child abuse. Diverse ethnicity with significant number of Native Americans. | Children: Described as newborns; Mothers: M=23.4 years. | 643 parent-child dyads. | | **Healthy Start Program:** 3 year intervention facilitated by paraprofessionals targeting ˅ risk factors child abuse and abuse prevention. | CESD-R and CAGE Substance Abuse Screening Test. |
| Duggan et al. (2009)  [ref 54] | RCT | USA/ Home | Mothers & children. Diverse ethnicity with significant number of Native Americans. Most were low SES. | Mothers: M=23.4, 23.7 (I, C) years. | 364 mothers and their children | | **Healthy Families Alaska:** 2 year paraprofessional intervention from birth-2 years facilitated by para-professionals targeting ^ engagement with EI services, ˄ parent-child relationship, ˅ parenting stress and ˅ maternal depression. | Infant-Toddler Home Observation for Measurement of the Environment (IT-HOME), CAGE Substance Abuse Screening Tool, Addiction Severity Index (ASI), NCAST, and case file data regarding maltreatment from Child Protection Services. |
| Dumas JE, Arriaga XB, Begle AM, Longoria ZN. Child and parental outcomes of a group parenting intervention for latino families: a pilot study of the CANNE program. Cult Divers Ethn Minor Psychol. 2011;17(1):107–15. https://doi.org/10.1037/a0021972. | Quasi-exp pre-post with f/u | USA/ Clinic | Ethnically diverse mothers of preschoolers recruited from daycares | Children: M=4.55 years; Mothers: M=28.4 years. | 124 parents | | **Criando a Nuestros Nin˜os hacia el E´xito (CANNE**), Spanish adaptation of **Parenting our Children to Excellence (PACE)**: parenting group targeting ˅child beh problems and ^ parenting skill. | SCBE and Behavior Assessment System for Children (BASC-2) and PPI. |
| Eckenrode et al. (2000)  [ref 86] | RCT | USA/ Home | Mothers and their infants. Significant number (47%) of mothers under 18 years of age. | Children: enrolled at birth. | 324 mothers | | Intervention facilitated by nurses during prenatal term and up to child’s 2nd birthday targeting ˅ child maltreatment. | CTS and review of child Protection Records to determine number of substantiated child maltreatment reports. |
| Eckenrode et al. (2001)  [ref 87] | Long-term f/u to RCT | USA/ Home | Sample of mothers recruited in prior study. Most were under 19, unmarried, low SES & African American ethnicity. | Children enrolled at birth. | 400 mothers | | **Elmira Nurse Home Visitation Program**: 2 year intervention targeting prevention of child beh problems. | Conflict Tactics Scale (CTS) and case file data regarding maltreatment from Child Protection Services. |
| Edwards et al. (2007)  [ref 36] | RCT | UK/ Community-based | Diverse SES parents of children at risk for conduct difficulties. | Children: M=45.98, 46.53 (I, C) months; Parent: M=30.16, 28.02 (I, C) years. | 116 parents (no gender breakdown given) | | **IY** (Basic): parenting group targeting ˅ child beh problems and ^ parenting skills. | ECBI. |
| Elliot et al. (2002)  [ref 135] | Long-term f/u to RCT | Australia/ Clinic | Mothers originally from non-English speaking countries including Vietnam, Malta, Philippines & Italy | Children: M=57 months. | 330 children and their parents | | **Sound Foundations:** parent group with individual sessions targeting ˅ child hyperactivity, ˅ anxiety, and ^ reading skills. | Peritraumatic Behavior Questionnaire, Parent completed (PBQ-P), PBQ-teacher completed (PBQ-T), Rutter Child Beh Questionnaire (RCBQ), teacher completed School Functioning Questionnaire. |
| Ericksen et al. (2018) [ref 196] | Feasibility study and Pilot RCT | Australia/ Hospital & Community-based | Mothers experiencing a range of postnatal mental health difficulties, including depression,  with infants of 12 months or less. | Feasibility study: Infant: M= 5.47 months, Mother: M= 31.52 years,  Pilot RCT Intervention group: Infant: M= 4.94 months, Mother: M= 32.31 years. | Feasibility study: 74 mother-infant dyads, Pilot RCT: 31 mother-infant dyads | | Community HUGS (CHUGS): ˅ associated problems in the mother−infant  Interaction. | Parenting Stress Index (PSI), Parenting Sense of Competency Scale  (PSOC; Self-Efficacy subscale), Paediatric  Infant Parent Exam (PIPE), Depression, Anxiety, Stress Scales (DASS), Edinburgh Postnatal Depression Scale (EPDS), program satisfaction. |
| Feinberg & Kan (2008)  [ref 128] | RCT | USA/ Clinic | Ethnically and SES diverse expectant parents. | Child: Included during prenatal period. No specific age data reported. Mothers: M=28.33 years; Father: M=29.76 years. | 169 couples | | **Family Foundations:** parenting group targeting ^ infant regulation, ^ co-parenting support, ^ parenting MH and ^ parent-child relationship. | CESD-R, Taylor Manifest Anxiety Scale (TMAS), Infant Behavior Questionnaire (IBQ). |
| Fergusson et al. (2005)  [ref 101] | RCT | Australia/ Home | Parents with diverse ethnicity and their children. Significant number of mothers/fathers of Maori ethnicity, single parents. Most on welfare, single parents, unplanned pregnancy. | Children: Range 3-36 months; Mother: M=24.6 years; Father: M=27.3 years. | 220 families | | 36 month nursing/social work targeting ^ child health, ˅ child abuse, ^ parental physical & mental health, ^ family economic well-being, and ˅ intimate partner violence. | Infant Toddler Social and Emotional Assessment Scale (ITSEA), The Child-rearing Practice Report (CRPR), PPI, CTS and review of health records, number of reports made to Child Protection Services. |
| Fernandez et al. (2011)  [ref 153] | One group pre-post | USA/ Clinic | Low SES mothers of African American ethnicity & their children. Balance of single parents, married, divorced. | Children: M=4 years, 5 months; Mothers (grandmothers): M=30 years. | 18 parent-child dyads | | **PCIT:** dyadic intervention targeting ˅ child beh problems, ˅ maternal depression and ˅ parenting stress. | ECBI, BDI, PSI, Peabody Picture Vocabulary Test (PPVT) and Wonderlic Personnel Test (WPT-Q). |
| Fisher, Rowe, & Feekery (2004)  [ref 121] | One group pre-post with f/u | Australia/ Residential setting | Caregivers of infants/preschoolers in foster care. | Children: Range 4-12 months. | 189 mothers and their children | | **Masada Private Hospital’s Mother Baby Unit:**  parenting group targeting ^ parent confidence & skills and ˅ mothers emotional issues. | Short Infant Temperament Questionnaires (SITQ), Barr Charts, Profile of Mood States (POMS-2), and EPDS. |
| Fleming et al. (2005)  [ref 102] | Quasi-exp pre-post with f/u | Canada/ Home | Low SES caregivers & children in Head Start. Most were mothers, with some grandmother. | Child: M=50 months; Caregiver: M=29 years. | 29 mothers and their children | | **Natural Teaching Strategies**: 10 sessions targeting ^ parental responsiveness and ^ engagement to the child. | Interactive Language Assessment Device and Turn Taking Coding System. |
| Fletcher (2009)  [ref 65] | Qualitative: Case study | Australia/ Home | One working class intact two parent family focus on father, but include his wife with PPD and child | Child: 3 months old at first home visit. | 3 fathers | | **Systematic Training in Effective & Enjoyable Parenting (STEEP):** 10 session video review parent-child interaction intervention targeting ^ parenting skills and ^ parental sensitivity to child’s cues. | Informal observation during intervention. Participants interviewed with video after intervention and videos transcribed and analyzed. |
| Fonagy, Sleed, & Baradon (2016)  [ref 197] | RCT | UK/ Hospital & Community- based | Parents with mental health problems who also were experiencing high levels of social adversity. | Children: Control: M= 3.8 months, Intervention: M= 3.9 months, Mother: Control: M=31.2 years, Intervention: M= 31 years. | 76 mother-infant dyads | | Parent-Infant Psychotherapy (PIP): improved infant development, parent-infant relational outcomes, and maternal mental health. | EAS, CIB, PDI, Parental RF, ARR, MORS, CES-D, BSI, GSI, PST, DC, P-CDI, PD, Self-Mastery Scale, ASQ-SE, SSP. |
| Ford, McDougall, & Evans (2009)  [ref 103] | RCT | UK/ Home | Low SES parents & children at-risk for school failure & in Sure Start. Most mothers were young single & Caucasian. | Children: M= 37, 36.7 (I, C) months. | 60 children and their mothers | | **Let’s Play in Tandem**: Weekly parent-child dyad sessions for 1 year targeting ^ child regulation and ^ child math/reading skills. | Four Counties Foundation Phase Profile of Academic & Social Skills, British Picture Vocabulary Scale (BPVS), British Ability Scales (BAS), Adapted inhibitory tests (i.e., day/night test, bear/dragon test, whisper test), and the Dimensional Change Card Sort (DCCS). |
| Franz, Weihrauch, & Schafer (2011)  [ref 66] | RCT | UK / Clinic | Depressed single mothers with preschool aged children | Mothers: 36.6, 34.4 (I, C) years. | 88 mothers | **PALME:** a preventative parent training program for single mothers targeting ˅ maternal depression, ^ maternal sense of parenting competence and ˅ child beh problems. | | Symptom Checklist 90- Revised (SCL-90-R), SF-36 Health Survey, Scales on the Experience of Emotions, Impairment Severity Score, Strengths and Difficulties Questionnaire (SDQ). |
| Fraser JA, Armstrong KL, Morris JP, Dadds MR. Home visiting intervention for vulnerable families with newborns: follow-up results of a randomized controlled trial. Child Abuse Negl. 2000;24(11):1399–429. https://doi.org/10.1016/S0145-2134(00)00193-9. | RCT | Australia/ Home | Low SES single parents ambivalent to pregnancy & history of family violence along with their children (vs control low SES teen parents with unstable housing with diverse marital status); most married. | Mothers: Range reported as a percentage of sample: sample: 15-17 years, 6.6%; 18-24, 31.5% and 25-41, 61.9%. | 181 mothers and their children | | 12 month nurse/ social worker intervention targeting ^ parenting skills, ^ parental reflective capacity, ˅ parent stress, ^ positive parent-child interactions, ^ access to resources and prevention of child abuse. | Child Abuse Potential Inventory (CAPI), PSI, EPDS, and the HOME. |
| Funderburk et al. (1998)  [ref 154] | One group pre-post with f/u | USA/ Clinic | Parents & their children with beh problems. Most were Caucasian. | Children: Range 2-7 years. | 12 children | | **PCIT:** 14 session targeting ˅ child beh problems at home & school. | ECBI, TRF, Sutter-Eyberg Student Inventory (SESBI),  Walker McConnell Scale of Social Competence & School Adjustment (SSCSA), Classroom Observation Coding System. |
| Gardner et al. (2007)  [ref 104] | RCT | USA/ Home | Low SES, highly stressed mothers children with conduct problems. Diverse ethnicity. Significant number of African American & biracial ethnicity & teen & single parents. | Children: M=23.7, 23.5 (I, C) months; Mother: M=26.2, 28.2 (I, C) years. | 120 mother-son dyads | | **Family Check-up:** 3 sessions based on motivational-interviewing principle targeting ˅ child beh problems, ˅ maternal depression and ˅ parent-child conflict. | CBCL, and in-home videotaped and coded observation of structured parent-child interactions. |
| Gardner et al. (2009)  [ref 37] | RCT | USA/Clinic | Low SES, highly stressed parents & children with conduct issues. Diverse ethnicity & significant number of teen & single parents. | Children: Range 2-4 years. | 731 mother-child dyads | | **Family Check Up:** 3 sessions based on motivational interviewing targeting ˅ child beh problems, parent-child conflict & maternal depression. | ECBI, CBCL,  Parenting Daily Hassles Scale (PDHS), Marital Adjustment Test (MAT), CESD-R. |
| Gardner et al. (2010)  [ref 38] | RCT | UK/ Clinic | Ethnically diverse, low SES, highly stressed parents of children at risk for conduct problems. High percent of teen-parents and single parents | Children: M=30 months. | 153 children and their mothers | | **IY** (Basic): parenting group targeting ˅ child beh problems. | ECBI, CBCL, CESD-R, PDHS, and MAT. |
| Gessner (2008)  [ref 105] | RCT | USA/ Home | Pregnant mothers & children. | Children: M=30.72 months. Mothers: Age reported as percentages of sample >20 years 34%, 20-29 years 51%, 29+ years 15%; Fathers >20 years 16%, 20-29 years 55%. | 985 children and their parents | | Paraprofessional & nursing intervention using across 2 years targeting child abuse prevention & ˅ referrals to child protection services. | Case file data regarding maltreatment from Child Protection Services. |
| Ghera (2009)  [ref 162] | RCT | Romania / Home | Romanian children from orphanages placed into foster care | Child: M=30.72 months | 136 children | **Foster care program** targeting ^ attention, ^ positive affect and ^ emotional expressiveness | | Observations from the Laboratory Temperament Assessment Battery (Lab-TAB). |
| Gill et al. (2008)  [ref 106] | Qualitative: Case Study | USA/ Home | One low SES Caucasian family. | Children: M=2 years; Mother: M=34 years; Father M =36 years. | 1 parent-child dyad | | **Family Check Up**: 3 sessions based on motivational interviewing targeting ˅ child beh problems, ˅ maternal depression and ˅ parent-child conflict. | ECBI, CBCL scale,  PDHS, MAT, and CESD-R. |
| Gjerdingen et al. (2009)  [ref 73] | RCT | Geographic location unreported / Clinic | English speaking mothers with PPD. | Children: 0-1 month; Mothers: 27.2, 28 (I, C) years. | 506 mothers | | Intervention targeting ˄ mother’s understanding of depression and ^ participation in treatment for depression. | Patient Health Questionnaire-9 (PHQ), Work Productivity Questionnaire, and parent self-reports regarding illness and days hospitalized. |
| Gormley et al. (2011)  [ref 107] | RCT | USA / School | Children from lower SES communities with parents of lower employment and education levels. | Children: All were 4 years of age. | 3166 children | | **Head Start-**based intervention targeting, ^ child adjustment to preschool, and ^ child beh at school. | Teacher reported Adjustment Scales for Preschool Intervention (ASPI) and the Instrumental Competence Scale for Children (COMPSCALE). |
| Gross, Fogg, & Tucker (1995)  [ref 129] | RCT | USA/ Clinic | Ethnically diverse and low SES parents of preschoolers with disruptive beh. | Children: M=29.9 months. | 46 parent-child dyads | | **IY:** parenting group targeting ˅ child beh problems; ^ parent attunement, ˅ parenting stress and ˅ parent depression. | CBCL, CESD-R, in-home videotaped parent-child interaction in structured activity coded and analyzed with the Relationship Process Code tool. |
| Guedeney et al. (2013) [ref 198] | RCT | France/ Home | Vulnerable primiparous mothers: planning to raise child alone, low SES, and/or having less than 12 years of schooling. | Children: 18 months, Mother: M= 22.3 years. | 367 mother-infant dyads | | Comp´etences parentales et Attachement dans la Petite Enfance:  Diminution des risques li´es aux troubles de sant´e mentale  et Promotion de la r´esilience (CAPEDP): improving child mental health, ˅ Postnatal Depression (PND) at 3 months postpartum, and optimiaing quality of home environment when child is 12 months old. | Full Alarm Distress Baby Scale (ADBB) Scale, The Modified ADBB (m-ADBB), Edinburgh Postnatal Depression Scale (EPDS). |
| Gwynne, Blick, & Duffy (2009)  [ref 148] | One group pre-post | Australia / Home & Clinic | Low SES parents and their children deemed to be at risk. | Children: Age data only given as percentage of the sample. | 23 caregivers and 42 children (no gender breakdown given for caregivers) | | **Spilstead Model** SM is a 12 month parent-child attachment based intervention targeting ˄ child education, and ^ in overall health and well-being of child. | CBCL, PSI, The Being a Parent Scale (adaptation of PSOC), Brigance Early Childhood Screens, North Carolina Family Assessment Scale (NCFAS-R). |
| Hahlweg et al. (2010)  [ref 125] | RCT | Germany/ Community-based | Diverse SES group of parents of preschoolers | Children: M=4.5 years; Mothers: M=35 years; Fathers: M=38 years. | 186 parents | | Parenting group targeting ˅ child beh problems and ^ parenting skills. | CBCL, TRF, PS, Positive Parenting Questionnaire, in-home videotaped and coded child-parent interactions coded using the Family Observation Schedule. |
| Harden et al. (2014) [ref 199] | Path analysis | USA/ Home | Low income mother-child dyads. | Children: Time 1: M= 16.68 months, Time 2: M= 25.92 months, Mother: M= 25.25 years. | 81 mother-child dyads | | Early Head Start: targeting ˅ maternal depression. | Bayley Infant  Neurobehavioral Screener (BINS), Brief Infant–Toddler Social and Emotional Assessment  (BITSEA), Parenting Stress Index Short Form (PSI/SF), Parent-Child Interaction Rating Scale (PCIRS). |
| Harnett & Dawe (2008)  [ref 115] | Quasi-exp pre-post | Australia/ Home or Clinic | Low SES families identified by social services as at-risk for child abuse. Most were single mothers | Children: M=4.4 years; Parent: M=32 years. | 10 parents (60% were mothers) | | **Parents Under Pressure (PUP) Program:** parent intervention targeting ˅ child beh problems, ˅ parenting stress, ˅ parenting depression & anxiety and prevention of child abuse. | SDQ, PSI, PDHS, DASS, CAPI, and Parental Support Questionnaire (PSQ). |
| Havighurst, Harley, & Prior (2004)  [ref 142] | RCT | Australia/ Clinic | Low/middle SES, ethnically & linguistically diverse parents of preschoolers. Most were intact families, unemployed with diverse parent education background. | Children: Range 4 - 5.11 years; Parents: M=36.52 years. | 218 parents (92% were mothers) | | **Emotion-focused parenting group:** parenting group targeting ˅ child beh problems, ˅ parent dismissiveness of child’s emotions and ^ parent competence & wellness. | ECBI, SDQ, b,  Coping with Children’s Negative Emotion Scale (CCNES), PSOC, Child Rearing Questionnaire, DAS, General Health Questionnaire (GHQ), and  Teacher reports on child’s social skills. |
| Havighurst et al. (2009)  [ref 143] | One group pre-post with f/u | Australia/ Clinic | Low SES parents of children with emotional/ beh difficulties. Most parents English speaking | Children: 4-5 years. | 218 parents (96% were mothers) | | **Emotion-focused parenting group:** parenting group targeting ˅ child beh problems, ^ child social skills, ˅ parent dismissiveness of child’s emotions and ^ parent competence. | ECBI, Difficulties in Emotion Regulation Scale (DERS), GHQ, and Maternal Emotional Style Questionnaire (MESQ). |
| Havighurst et al. (2010)  [ref 88] | RCT | Australia/ Clinic | Parents of diverse SES and educational background. Most were mothers who were cohabitating. | Children: Range 4-5 years. | 216 parents (96% were mothers) | | **Emotion-focused parenting** **group**: parent training group targeting ˅ child beh problems and ˅ parent’s dismissiveness of child’s emotions. | DERS, MESQ, Parenting Emotional Style Questionnaire (PESQ) and in-home videotaped and coded structured task. |
| Henry GT, Henderson LW, Ponder BD, Gordon CS, Mashburn AJ, Rickman DK. Report of the findings from the early childhood study: 2001–02. Atlanta: Georgia State University School of Policy Studies; 2003. | Quasi-exp pre/post (3 group) | USA / School | Children enrolled in Georgia's universal Pre-K program, Head Start program, or private preschools (comparison group). Diverse ethnicity of Caucasian & African American. Significant number of children born to teen moms. | Children: M=4.5 years. | 466 children | | Preschool programming offered over 180 days targeting ˄ school readiness (language, spatial, and colors) and ˄ socio-emotional functioning. | PPVT, WJ III ACH, Comprehensive Test for Phonological Processing (C-TOPP), CIS, and Quality of Environment Observation measure. |
| Hiscock H, Bayer JK, Price A, Ukoumunne OC, Rogers S, Wake M. Universal parenting programme to prevent early childhood behavioural problems: cluster randomised trial. BMJ. 2008;336:318. https://doi.org/10.1136/bmj.39451.609676.AE. | Clustered RCT | Australia/ Clinic | Parents of children from diverse SES. Most were married/ cohabitating. | Children: Range 1-42 months; Mother: M=33 years. | 733 mothers | | Parenting intervention facilitated by well-child providers and parent experts targeting ˅ child beh problems, ^ parenting skills and ^ parenting MH. | CBCL, PBC and DASS. |
| Hoffman et al. (2006)  [ref 108] | One group pre-post | USA/ Clinic | Low SES parents & their preschoolers. Most were single Caucasian parents. | Children: M=32 months; Parents; M=23.8 years. | 65 parent-child dyads | | **Circle of Security:** 20 weekly sessions targeting ^ attachment. | SSC. |
| Horwitz et al., 2014  [ref 200] | RCT | USA/Palo Alto, CA | Mothers of premature infants with symptoms of trauma, anxiety, or depression. | M=33.8  Infant Gestational Age=30.6 weeks | 98 Mothers | | 6-9 session, trauma-focused CBT intervention including perceptions of vulnerability | Vulnerable Baby Scale (VBS) |
| Hourihan & Hoban (2004)  [ref 149] | Prospective single group repeat measures | UK/ School & Community-based | Rural parents of children with conduct difficulties. | Children M=3.6 years. | 33 children and their parents (no parent gender breakdown) | | **IY:** parenting group targeting ˅ child beh problems with concurrent and **Kids Challenge & Change** program targeting ^ child social skills. Both programs targeting prevention of child beh problems. | Wilcoxon Signed Ranks Test, Merill’s Preschool and Kindergarten Behaviour Scales Not specified. |
| Huang et al. (2003)  [ref 144] | Quasi-exp pre-post | China/ Clinic | Parents of children with ADHD. Most were college educated & employed. | Children: M=65 months; Parent: M=35.2 years. | 23 parents (96% were mothers) | | **Barkley-based Parenting** group with booster session targeting ˅ child beh problems and ˅ ADHD symptoms. | Disruptive Behavior Rating Scale (DBRS), Child Attention Profile, and HSQ. |
| Huber, McMahon, & Sweller, 2015  [ref 201] | Quasi-exp pre-post |  | Parent-child dyads. | Children: M=47.80 months; Parent age not reported | 83 children & 83 parents | | **Circle of Security:** 20 weekly sessions targeting ^ attachment. | Strange Situation Procedure |
| Hustedt et al., 2017  [ref 202] | Quasi-exp pre-post | USA / Home/ Community-based | Poverty/At-Risk Families in EHS program | Children: M=19.2 months; M=29.1 years | Children: M=84; Parents: M=84 | | Early Head Start (EHS): ^ cognitive, social, and emotional development  Child, ^parent1 involvement. | FAD, CES-D, GAD-7, ECR-S, PSI/SF, Child Cortisol Levels, and BITSEA. |
| Hutchings (1996)  [ref 49] | Quasi-exp pre-post with f/u | UK/ Community-based | Mothers on income support with preschoolers with conduct difficulties. Most parents were Welsh speaking. | Children: M=2.4 years; Mother: M=24 years. | 7 Mothers | | Parent group with follow-up sessions targeting ˅ child beh problems and ^ maternal MH. | ECBI completed by Health Visitor, parent reported GHQ, and PSI. |
| Hutchings et al. (2007)  [ref 39] | RCT | UK/ Community-based | Parents of children at risk for conduct disorder. Balance of cohabitating & single parents; significant number of low SES and Welsh speakers. | Children: M=46.4 months; Mother: M=21.4 years. | 153 parents | | **IY** (Basic): parenting group targeting ˅ child beh problems. | ECBI, PSI, BDI, and home-based observation with DPICS-III. |
| Huxley P, Warner R. Primary prevention of parenting dysfunction in high-risk cases. Am J Orthopsychiatry. 1993;63(4):582–8. https://doi.org/10.1037/h0079478. | Quasi-exp pre-post | USA / Home | High risk mothers in third trimester of pregnancy | Mothers: M=20 years | 40 mothers | **Community Infant Project**: a tri-agency, interdisciplinary early intervention targeting ˅ involvement with child protective services, v substantiated cases of child abuse/neglect. | | Adult/Adolescent Parenting Inventory (AAPI), HOME, and case file data regarding maltreatment from Child Protection Services. |
| Irueste-Montes & Montes (1988)  [ref 116] | Quasi-exp pre-post | USA / Home & School | Participants were families with documented cases of abuse and neglect who were referred by El Paso County Department of Social Services. | No mean age data provided. | 42 families (75% were mothers) | **R&R Treatment Program:** including therapeutic family day care, parenting group and in-home treatment for the entire family targeting ^ parenting competence and ˅ criticism of child. | | Observation using a behavioral observation coding system. |
| Joachim et al. (2010)  [ref 145] | RCT | Australia/ Clinic | Urban, highly educated parents of children with disruptive  beh problems. | Children: M=3.23 years; Parents: M=33.78 years. | 46 parents (96% were mothers) | | **Positive Parenting Program (Triple P):** parenting group targeting ˅ child beh problems, ^ parenting confidence, ^ parenting skills and ˅ parent anxiety & depression. | ECBI, parent reported Shopping Observation Checklist, PS, Parenting Task Checklist (PTC), and DASS. |
| Johnson & O’Fallon (1974)  [ref 30] | One group pre-post | USA / Home & Community-based | Children in rural Tennessee who would not otherwise be able to attend preschool. | Children: Range 3-5 years. | 335 children and their parents. | | **Clinch Powell Educational Cooperative:** preschool programming delivered via TV by van within the community over a 33 week in conjunction with weekly home-based activities period targeting ˄ social skills, ˄ positive self-concept, ^ cognitive abilities and ˄ parents in education process. | The Behavior Assessment Interview (BAI). |
| Jones et al. (2007)  [ref 40] | RCT | UK/ Community-based | Mothers of children with early signs of conduct problems and ADHD. Most were single mothers. | Children: M=46.50 months; Mother: M=27.54 years. | 133 mothers | | **IY** (Basic): parenting group targeting ˅ child beh problems, ˅ ADHD symptoms and ˅ conduct difficulties. | ECBI, SDQ, Connors Abbreviated Parent/Teacher Rating Scale (ATRS), and observation with DPICS-III. |
| Julian et al. (2018)  [ref 211] | Quasi-exp pre-post | USA/ Group & Home | Military families with young children enrolled in SMF, mostly Caucasian with some college. | Parents: Range 22 to 40. | 78 parents (46 mothers & 29 fathers) | | **Strong Military Families (SMF):** home-based psychoeducation & multifamily therapeutic group aimed to, ^ parental self-care, ^ positive parenting, ˅ parental stress, ^ social support. | WMCI, WMCI Parenting Reflexivity Scale. |
| Kaminski et al. (2002)  [ref 109] | RCT | USA/ Home, school & community-based. | Parents and their children: Most on income support and majority of Caucasian ethnicity. | Children: M=4 years. | 147 children and their parents | | Parent and teacher education programming provided across a 2 year period targeting ˅ risk of the child’s future substance abuse, ^ social competence, ^ school bonding and ^ caregiver self-regulation. | CBCL, ECBI, Social Competence Scale, PPI, teacher reported Parent-Teacher Involvement Questionnaire (INVOLVE-T), and videotaped recorded observation of parent-child interaction coded with McMahon's Beh Coding System. |
| Kennedy, Rapee, & Edwards (2009)  [ref 31] | RCT | USA/ Clinic | Middle-upper SES, parents with anxiety of inhibited children. Most parents of Anglo-Celtic ethnicity. | Children: M=47.07 months. | 71 children and their parents (94% mothers) | | **Cool Kids**: parenting group targeting ˅ child anxiety and ˅ parent anxiety. | Short Temperament Scale for Children (STSC), Behavioral Inhibition Questionnaire (BIQ), Preschool Anxiety Scale, Child Anxiety Life Interference Scale (CALIS), DASS. Beh observation in laboratory at pre and post. |
| Kersten-Alvarez et al (2010)  [ref 169] | RCT | Netherlands/ Home | Mothers & children. Most were of Dutch ethnicity with diverse education and SES. | Children: M=67.6 months; Mothers: M=35.7 years. | 85 parent-child dyads | | Intervention provided by parenting specialists 8-10 sessions targeting ^ quality of mother child interaction and ^ attachment. | CBCL, Adapted version of the Attachment Story Completion Task (ACST), Puppet Interview, Teacher completed California Child Q-Set, Preschool Social Behaviour Questionnaire (PBQ), and Teacher School Adjustment Stress Response Scale, Caregiver-Teacher Report Form and video recorded observations of maternal-child interaction. |
| Keys et al., 2013  [ref 203] | Meta-Analysis | USA/ Pre-school programs | Children across various studies from various care centers |  | 733, 1,300, 2,898, 1,140 | | Psychoeducation on childhood development, health & learning experiences, Early Head Start | PPVT-III, WJ, CPSC, TCRS, CBCL, BSID, CBCL, & TCRS conduct problem subscale. |
| Klein Velderman et al. (2006)  [ref 59] | RCT | Netherlands/ Home | Mothers with insecure attachment & their infant. | Children: M=6.83 years; Mothers: M=27.8 years. | 81 parent-child dyads | | **Video Taped Feedback (VIPP)** and **Enhanced Video Taped Feedback (VIPP-R)** 5 sessions targeting ˅ child beh problems, ^ maternal sensitivity to infant cues, ^ maternal attachment and ˅ maternal stress. | CBCL (Dutch translation), IBQ, SSC, non-standardized measure of parent social support and video recorded observation of mother-child interaction coded with Ainsworth’s maternal sensitivity protocol. |
| Kotler & McMahon (2004)  [ref 136] | RCT | USA/ Research Laboratory setting | Mothers & their preschoolers who were anxious vs angry vs socially competent. Most were of Caucasian ethnicity. | Children: M=4.29 years. | 60 parent-child dyads | | **Child’s Game Training Protocol:** skill & practice-based sessions targeting ^ child compliance, ^ mother’s attention to child cues and ^ parenting skills. | Videotaped coded observation of parent-child interaction during play with instructional tasks. |
| Lally (1987)  [ref 89] | One group pre-post with f/u | USA/ Home & Daycare | Young mothers of low SES recruited in last trimester. Most were of Black ethnicity & most were single parents. | Children: Range 18-60; Mothers: M=18 years. | 82 mothers and their children | | **Syracuse University Family Development Research Program**: extended duration parent intervention with child involved in-home component targeting ^ child school functioning, ^ parenting skills and ^ parent-child interaction. | SB and observer rating of child’s social-emotional functioning. |
| Landsem, Handegard, Ulvund, & Tunby (2015)  [ref 204] | RCT | Netherlands/Hospital & In-Home | Preterm infants born at the University Hospital of Norway | Children:  Range <28 weeks – 9 years | 221 Children | | **Mother-Infant Transaction Program:** Seven 1-hour sessions with parents and baby focused on strengthening. | Child Behavior Checklist (CBCL) by parents & Strengths & Difficulty Questionnaire (SDQ) on Teacher Report Form (TRF) |
| Landy, Menna, & Sockett-Dimarcio (1997)  [ref 41] | Quasi-exp pre-post | Canada / Clinic | Mothers of children aged 3-6 with beh problems and noncompliance. | Children: M=4.75 years. | 25 mothers | **HEAR: Helping Encourage Affect Regulation:** parenting group targeting ˅ child beh problems and ^ parenting capacity | | CBCL, Emotional Availability Scales (EAS), Quality of Instruction Scale. |
| Lange et al. (2018)  [ref 205] | RCT | Denmark/ Clinic | Children with ADHD | Children: Range 3-7 years | 164 Children | | **New Forest Parenting Programme**: parent training program ˅ child behavior problems, ^ parenting capacity, ˅ parenting stress. | Parent report on ADHD-RS-IV-Preschool Danish Version, Teacher ADHD RS-IV, Parenting Sense of Competence Scale, FSI, Adult ADHD Self-Report Scale. |
| Lavigne et al. (2008)  [ref 137] | RCT | USA/ Clinic | Parents of children with beh problems. Most were middle class, Caucasian and married. | Children: M=4.6 years. | 117 parents | | **IY:** parenting group targeting ˅ child beh problems. | ECBI, CBCL, Rochester Adaptive Battery Inventory (RABI), Children’s Global Assessment Scale (CGAS), PSI, BDI, and video recorded observation of parent-child interaction. |
| Lavigne et al. (2010)  [ref 138] | Quasi-exp pre-post | USA/ Clinic | Parents of children with beh problems. Most were middle class, Caucasian and married. | Children: Range 4-6.11 years. | 86 parents | | Parent training facilitated by nurses and psychotherapists targeting ˅ child beh problems. | ECBI, CBCL, RABI, PSI, BDI, PPVT, CGAS, video recorded observation of parent-child interaction. |
| Leifer et al. (1989)  [ref 60] | Single-case control | USA/ Home & Clinic | One mother & her infant. | Children: 2 months. | 1 parent-child dyad | | **Parent-infant relationship therapy (PIRT):** targeting ^ maternal confidence and ^ parent-child attachment. | Bayley-III, SSC, Life Stress Scale, The HOME, video recorded observation of parent-child interactions. Arizona Social Support Interview Schedule (ASSIS), and maternal interview regarding functioning. |
| Lecannelier Silva, & Hoffmann (2014)  [ref 206] | Quasi-exp pre-post | Chile / State-run Centers | Institutionalized infants & their caregivers. | Infants: Range 2-12 | Infants: 62 | | “parental sensitivity manual”: 2-month training course targeting ^ parent-infant interaction, ^ interactive play, ^ attachment | Massie-Campbell’s Attachment During Stress scale (ADS), psychomotor developmental scale (EEDP), Infant Behavior Record (IBR) |
| Lenze, Pautsch, & Luby (2011)  [ref 146] | One group pre-post | USA/ Clinic & home | Parents and children with depressive symptoms: Diverse ethnicity and SES. Most parents were married. | Children: M=4.38 years. | 8 parent-child dyads (75% of parents were mothers) | | **PCIT:** dyadic sessions with home-based follow-up targeting ˅ child depression symptoms, ^ emotion recognition and differentiation skills. | Health Beh Questionnaire (HBQ),  Preschool and Early Childhood Functional Assessment Scale (PECFAS) , BDI, Parent Acceptance Inventory (PAI), Therapy Attitude Inventory (TAI), Penn Emotion Differentiation Test. |
| Lester et al. (2016)  [ref 207] | Quasi-exp pre-post | USA & Japan / Military Installations | Military families & children with high levels of stress related to parental wartime military service | Children: 7.26 years @ intake; Parents: 33.4 years @ intake | 2,615 families (1,426 service member parents, 2,073 civilian parents, and 3,810 children). | **Families Over Coming Under Stress (FOCUS):** family-centered preventative program aimed to ^ resilience and ˅ psychological health risk in military families | | Brief Symptom Inventory-18 (BSI-18), SDQ – Parent Report, FAD, PTSD Checklist, MASC, KidCope |
| Leung, Tsang, & Dean (2011)  [ref 150] | RCT | China / Clinic | New immigrant parents in Hong Kong. | Children: M=4.6 years; Mothers: M=32.48 years; Fathers: M= 38.77 years. | 120 parents (no gender breakdown given for parents) | **HOPE: Hands On Parent Empowerment:** parenting education group targeting ˅ child beh problems, ^ parenting confidence, ˅ parenting stress and ^ parent sense of social support. | | ECBI, PSI, Duke-UNC Functional Social Support Questionnaire and General Self Efficacy Scale (GSE-10). |
| Levac et al. (2008)  [ref 48] | Qualitative: Content Analysis | Canada/ Clinic | Ethnically diverse parents. Balance of married & single parents with a range of education and SES. | Children: Range 4-12 years; Parents: M=40 years. | 41 parents | | **IY:** parenting problems targeting ˅ child beh problems, ^ parenting skills, confidence & stress, and ^ parent-child relationship**.** | Semi-structured interviews with parents used to determine their perceptions of IY’s effectiveness. |
| Leve et al. (2009)  [ref 74] | Prospective cohort design | USA / Clinic | Children and their adoptive and biological parents. | Children: M=9 months; adoptive Mothers: M=37 years; adoptive Fathers: M= 38 years; Birth Mothers: M=24 years; Birth Fathers: M= 25 years. | 290 linked triads (child, adoptive parent, and birth mother) | **Structured Parenting Program:** targeting ˅ child beh problems | | CBCL, Elliot Social Behavior Questionnaire, BDI, Beck Anxiety Inventory, Composite International Diagnostic Interview Alcohol Drug Dependence Scale, Parent-Child Free Play and Compliance Task Coding Manual. |
| Lieberman et al. (1991)  [ref 67] | RCT | USA/ Home & Clinic | Low SES Spanish speaking Latino mothers & their infants with anxious attachment. | Children: Range 11-14 months; Mothers: Range 21-30 years. | 100 parent-child dyads | | Weekly parent-child psychotherapy sessions for 1 year targeting ^ maternal response to infant cues and ^ attachment. | SSC, Attachment Behaviour Q-Sort, Life Events Inventory (LEI), Maternal Attitude Scale (MAS), videotaped observation of free play sessions coded for mother-child attachment beh. |
| Lieberman AF, Ghosh Ippen C, Van Horn P. Child-parent psychotherapy: 6-month follow-up of a randomized controlled trial. J Am Acad Child Adolesc Psychiatry. 2006;45(8):913–8. https://doi.org/10.1097/01.chi.0000222784.03735.92. | RCT | USA/ Clinic | Mothers & children exposed to violence. Diverse ethnicity, most were Latino. | Children: M=4.06 years. | 125 parent-child dyads | | 50 sessions of parent-child psychotherapy targeting ˅ child beh problems and ˅ child PTSD symptoms. | CBCL and SCL-90-R. |
| Lieberman AF, Van Horn P, Ippen CG. Toward evidence-based treatment:child-parent psychotherapy with preschoolers exposed to marital violence. J Am Acad Child Adolesc Psychiatry. 2005;44(12):1241–8. https://doi.org/10.1097/01.chi.0000181047.59702.58. | RCT | USA / Clinic | Mother-child dyads previously exposed to intimate partner violence. | Children: M=4.06 years. | 50 parent-child dyads | **Child Parent Psychotherapy:** intervention targets ˅ child beh problems, ˅ child trauma symptoms, and ˅ maternal trauma symptoms. | | CBCL, SCL-90-R, Clinician Administered PTSD Scale. |
| Long et al. (1994)  [ref 176] | Long-term f/u to RCT | UK/ Clinic | Parents previously in a parenting group & their children. | Children: Range 2-7 years; Parents: M=20 years. | 26 parents (65% of parents were mothers) | | 8-10 educational/ modeling sessions with parents targeting ˅ child beh problems and ^ parenting skills. | Conflict Behaviour Questionnaire (CBQ), Brief Symptom Inventory (BSI), National Youth Survey (NYS) on drug use, delinquency, Rosenberg Self Esteem Scale (RSES) and child’s academic grade. |
| Love (2010)  [ref 110] | RCT | USA / Home | Low income families with children under 3 years of age. | Child: M=24.95 months. | 3001 families | **Early Head Start**: a home visiting program prerequisite to the Head Start Preschool targeting ^ child cognitive & language development, ˅ child aggressive beh and ^ family use of community services. | | CBCL, Family Adaptability and Cohesion Evaluation Scale (FACES), Lieter Scaled Attention, PPI, CIS, and the HOME scale. |
|  |  |  |  |  |  |  | |  |
| Lowell et al. (2011)  [ref 42] | RCT | USA / Home & Community-based | Low SES mothers and their children living in inner-city settings. Most single and of Latino/ Hispanic & African American ethnicity and significant % of partners with history of incarceration. | Children: Range 6-36 months. Mothers: M=27.7 years. | 157 mothers and their children | | **Child FIRST** (Family Interagency, Resources, Support and Training): a team-based intervention matching the client’s culture/language over 45-90 sessions targeting prevention or ˅ in child emotional disturbance and/or developmental or learning disabilities and ˅ in child abuse and neglect. | ITSEA, BSI, CESD-R. |
| Luby, Lenze, & Tillman (2012)  [ref 158] | RCT | USA/ Clinic | Parents & their children with depressive symptoms. Most in intervention condition were of Black ethnicity with diverse SES. | Children: Range 3-7 years. | 157 parent-child dyads (most parents were mothers) | | **PCIT**-based program, **PCIT-Emotion Development** targeting ^ child executive functioning and ˅ parenting stress. | HBQ, PECFAS, ERC, Behavior Rating Inventory of Executive Function–  Preschool Version (BRIEF-P), BDI, and Penn Emotion Differentiation Test. |
| Lyons-Ruth K, Connell DB, Grunebaum HU, Botein S. Infants at social risk: maternal depression and family support services as mediators of infant development and security of attachment. Child Dev. 1990;61(1):85–98. https://doi.org/10.1111/j.1467-8624.1990.tb02762.x. | Quasi-exp pre-post | USA/ Home & Community-based | Low SES depressed mothers & children: Most on government assistance. Significant number of ethnic minority mothers. | Children: M=4.7 months; Mothers: M= 25.6, 21.8 (I, C) years. | 31 parent-child dyads | | Parenting program over 9-18 months alternating parent and MH professional visitors within the home working with mother-child dyad targeting ^ parent-child relationship, ˅ parental isolation and ^ access to resources. | SSC, Bayley-III, CESD-R, and interviews with mother regarding their social isolation. |
| Maguin et al. (1994)  [ref 68] | RCT | USA / Clinic & Home | Families with preschool aged boys and alcoholic fathers. | Children: M=4.4 years; Mothers: M=29.3 years; Fathers: M= 31.4 years. | 81 families | **Michigan State University Multiple Risk Child Outreach Program:** Parent training program combined with marital counseling targeting ˅ child ext beh, ^ prosocial beh, and ^ child affectionate beh. | | CBCL, Conners Parent Questionnaire - Modified, Child Behavior Rating Scale - Preschool Edition (CBRS-P) and Parent Daily Report. |
| Matos, Bauermeister, & Bernal (2009)  [ref 130] | RCT | Puerto Rico/ Clinic | Puerto Rican Spanish speaking & their children with ADHD. | Children: Range 4-6 years. | 32 parent-child dyads. | | **PCIT** dyadic intervention: targeting ˅ child beh problems, ˅ ADHD symptoms and ^ parent/family functioning. | ECBI, BASC-2, DBRS (Spanish/parent), CGAS, PPI and BDI (Spanish). |
| Matthey et al. (2004)  [ref 119] | RCT | Australia / Clinic | Fathers and pregnant mothers most with a high school education, convenience sample recruited from participants in routine antenatal care. | Mother: M= 27.1 years; Father: M=29 years. | 268 parents | | 6 session psychosocial group intervention targeting the prevention of PPD. | Diagnostic Interview Schedule for Children (DISC), SCID, EDPDs, Coppersmith Self-Esteem Inventory (CSEI), POMS-2, CESD-2, Significant Others Scale (SOS), PSOC, and Partner Awareness Scale. |
| Mayers et al. (2008)  [ref 58] | Quasi-exp pre-post with f/u | USA / School | Low SES teen mothers attending inner city high schools & their infants in school’s daycare. Most were of African-American ethnicity. | Children: M=11 months; Mothers: M=17 years. | 52 parent-child dyads | | **Chances for Children:** 6-20 month dyadic intervention mixing psychodynamic sessions for mothers with dyadic & parent group **Partners in Parent Education** targeting ^ maternal responsiveness and ^ maternal affective availability. | Infant Characteristics Questionnaire (ICQ), CESD-R, PSI, and videotaped parent-child free play coded with Maternal Behavior Rating Scale (MBRS). |
| McCabe & Yeh (2009)  [ref 50] | RCT | USA/ Clinic | Mexican- American, Spanish speaking & their children with clinical beh problems. | Children: Range 3-7 years. | 58 parent-child dyads (92% of parents were mothers) | | **GANA Program:** cultural version of **PCIT** for Mexican-Americans targeting ˅ child beh problems; ^ parent-child relationship. | ECBI, CBCL, Early Childhood Inventory (ECI-4), PPI, PSI, and videotaped parent-child interactions coded with DPICS-III. |
| McGilloway et al. (2012)  [ref 51] | RCT | Ireland/ Community-based | Low SES parents of children with beh problems. | Children: M=59, 55.2 (I, C) months. | 149 parents (96% of parents were mothers) | | **IY** (Basic): Parenting group targeting ˅ child beh problems, ˅ child ADHD symptoms, ^ child social skills, ^ parenting skills, ˅ parenting stress and ^ parent well-being. | ECBI, SDQ, CAPRS, SCS, PSI, BDI, and videotaped parent-child interactions coded with DPICS-III. |
| Melhuish et al. (2008)  [ref 95] | Quasi-exp | UK/ Home | Families living in disadvantaged Sure Start Areas with children aged 3 years. | Children: M=3 years. | 5883 families | **Sure Start Local Programs:** intervention targeting ˅ social inequalities, ^ child physical health, ^ child language abilities, ^ child prosocial beh, ^ maternal well-being, ^ parenting capacity and ^ family functioning | | BAS, the HOME scale, and parent reports of primary care services. |
| Mihelic, Morawska, & Filus (2018)  [ref 208] | RCT | Queensland / Group & Telephone-based | Fathers expecting their first baby | Fathers: 34.25 years | 112 Fathers | | **Baby Triple P:** aimed to improve paternal psychological distress, ^ paternal confidence, ^ paternal competence, ^ levels of relationship happiness & life satisfaction | The Edinburgh Postnatal Depression Scale (EDPS), Depression Anxiety Stress Scale (DASS), Oxford Happiness Questionnaire (OHQ), Maternal Self-Report Inventory (MSRI), Maternal Self-Efficacy Scale (MSES), PAFAS, Household and Childcare Task Checklist, RQI. |
| Milford et al. (2006)  [ref 151] | Quasi-exp pre-post with f/u | UK/ Home | Parents & children with sleeping, eating or beh problems | Children: Described only as infants. | 22 parents and their children | | **Solihull Approach:** paraprofessional intervention targeting ˅child beh problems, ^ parenting skill and ^ positive parent-child interactions. | PSI and Visual Analogue Scales (VAS). Defining perceived severity of the problem. |
| Milgrom et al. (2006)  [ref 75] | Long-term f/u to RCT | Australia/ Clinic | Mothers with PPD & their infants. | Parents: M=31.96, 32.63 (I, C) years. | 22 dyads | | **Happiness, Understanding, Giving and Sharing (HUGS):** a manualized 3 session mother-infant program added to 12 sessions of CBT targeting ^ quality of mother-infant. | PSI and BDI. |
| Milgrom et al. (2011)  [ref 76] | RCT | Australia / Telephone-based | Mothers at risk of PPD. | Mothers: M= 31.96, 32.63 (I, C) years. | 143 mothers | | **Towards Parenthood:** intervention targeting the prevention of PPD, ˄ develop awareness and skill for transition to parenting, and ˅ parenting stress. | EPDS, DASS, BDI, and PSI. |
| Miller et al. (2014)  [ref 209] | Quasi-exp pre-post | USA/Community-based | Children from homes with high, middle, and low levels of parental pre-academic stimulations | Children: Range 3-4 years | 3,185 Children | | **Head Start:** 36 -month parent-focused para-professional intervention targeting ^ child cognitive skills and ^ attachment. | Woodcock-Johnson (WJ) III Applied Problems (early math, early literacy, receptive vocabulary) |
| Morawska & Sanders (2006)  [ref 131] | One group pre-post | Australia/ Home | Parents who self-reported their toddler’s behaviour problems. Most were employed, middle income, 71.8% two-parent households. | Children: M=26.63 months; Mother: M=31.90 years; Fathers: M=34.72 years. | 110 parents | | **Behavioural Family Intervention (BFI)** based on *Every Parents Self Help* book and video with 10 weekly phone sessions by counsellors targeting ˅ child beh problems and ^ parenting efficacy. | ECBI, Parental Anger Inventory, PS, Relationship Quality Index, and DASS. |
| Morgan et al. (2017)  [ref 210] | RCT | Australia / Online | Parents of children aged 3-6 years with an inhibited temperament | Parent: M=35.9 | 433 parents | | **Cool Little Kids:** Six group online sessions ^ child coping, ˅ parental overprotection, ^ child independence | STSC, PAS-R, OAPA, SDQ-P (parent version), Life Interference Scale (CALIS-PV), Over-involved/Protective parenting scale (OI/P), Psychological Distress Scale (K10) |
| Morrell et al. (2009)  [ref 120] | Clustered RCT | UK/ Clinic | English speaking mothers with diverse first languages & their children. Most were Caucasians living with partner. | Mothers: M=31.3 years. | 3436 mothers | | Intervention provided by health visitor targeting ^ identification of PPD. | EPDS, CORE Outcome Measure, Measure of Social Relationships (MSR), Life Events Questionnaire (LEQ), PSI, DAS, and State-Trait Anxiety Inventory (STAI). |
| Morrison & Bratton (2010)  [ref 43] | RCT | USA / School | Headstart educators working with lower SES children of diverse ethnic/cultural backgrounds including Hispanic, Black, and White. | Children: All under 5 years but no specific age reported. Teachers: M=37 years. | 24 Teachers; 52 children. | | **Child-teacher Relationship Training (CTRT):** a structured training program targeting ^ educator participation in early MH interventions and ˅ child beh problems. | CBCL and TRF. |
| Moss et al. (2011)  [ref 117] | RCT | Canada/ Home | Low SES French-speaking parents involved with child welfare & their children at-risk for maltreatment. Most were single parents. | Children: Range 1-5 years. | 67 parent-child dyads. (94% of parents were mothers) | | 8 sessions of attachment training with parent-child dyad using video tape review targeting ^ parental sensitivity and ^ attachment. | CBCL and Maternal Behaviour Q-sort (MBQS). |
| Nanzer et al. (2012)  [ref 77] | Quasi-exp pre-post | Switzerland / Clinic | Mothers with perinatal depressed. | First treatment pre-child birth through 9.4 weeks post-delivery. Mothers: M=33 years. | 40 mothers | | Four sessions of individual psychotherapy targeting ˅ parent depression and ˄ child-parent interaction. | EPDS, DADP, Global Assessment of Functioning (GAF), and Parent-infant Relationship Global Assessment Scale (PIR-GAS). |
| Newman & Stevenson (2008)  [ref 78] | Qualitative: Case study | Australia/ Clinic | Mothers with borderline personality disorder & their children. Recruited while pregnant. | Child: 2 years; Mother: 35 years. | 2 mothers | | **Watch, Wait & Wonder**: 12-14 session manualized parent-infant intervention beginning prenatally targeting ^ maternal sensitivity and ^ attachment. | Qualitative study. |
| Nicholson et al. (2010)  [ref 122] | Quasi-exp pre-post | Australia/ Clinic | Marginalized mothers and fathers of birth to three year old children. | Children: M=26.8 months; Parents: M=31.84 years. | 850 parent-child-dyads (no gender breakdown given for parents) | **Sing and Grow:** a 10-week group music therapy intervention targeting ^ child development and ^ positive parenting. | | Parent Scales adapted from the Longitudinal Study of Australian Children and in-session observation of parent-child interaction. |
| Nixon et al. (2004)  [ref 155] | Long-term f/u to RCT | Australia / Clinic vs. Home | English speaking parents & children with beh problems. | Children M=47.36, 48.30 (I, C) months; Parent M=34.73, 33.85 (I, C) years. | 54 dyads | | **Parent-Child Interaction Therapy (PCIT):** 12 sessions of PCIT versus abbreviated sessions with video review and phone consult targeting ˅ child beh problems. | ECBI, CBCL, and videotaped parent-child interactions coded using the DPICS-III. |
| Novins, Ferron, Abramson, & Barlow (2018)  [ref 212] | Descriptive | USA / Tribal home-based | Perinatal and early childhood home-visiting programs  serving tribal communities funded through federal Tribal MIECHV |  | 9 tribal home-visiting program | | **Tribal Maternal, Infant, and Early Childhood Home Visiting Program (Tribal MIECHV):** multiple programs focused on various aspects of parent-child relationship; this study focused identifying programs that address substance use issues within the home, with aims to ^ detection of substance use problems and ^ competency in relapse prevention | Survey focused on identifying program approaches to addressing substance-use services in their home-visiting services |
| Nye et al. (1995)  [ref 69] | RCT | USA/ Clinic and Telephone-based | Mid-SES families or preschoolers with an alcoholic father. Most were Caucasian. | Children: M=4.4 years; Mother: M=30.1 years; Father: M=32.2 years. | 42 families | | **Oregon Social Learning techniques and Parent Training**: 28 session family-based educational program targeting ˅ child beh problems. | CBRS preschool version, BDI, The Hamilton Rating Scale (HRSD) and CTS. |
| Nye, Zucker, & Fitzgerald (1999)  [ref 44] | RCT | USA / Clinic | Lower SES families, where the father is dealing with alcoholism and their children. | Children: Range 3-5 years; Mothers: M=29 years; Fathers: M=32 years. | 52 families | | Intervention targeting ˅ child beh problems, ˅ social aggression and ^ parenting skill. | CBCL-Preschool Edition and Observer Impressions Inventory. |
| Ogg et al. (2014)  [ref 213] | Quasi-exp pre-post | USA / Community-based | Parents of young children who attended a behavioral parent-training program, delivered English & Spanish, about half Caucasian, about 1/3 Hispanic | Parents: M=34.8 | 739 parents | | **Helping Our Toddlers Developing Our Children’s Skills (HOT DOCS**): parent-training program targeting ˅ child beh problems, ˅ parental stress, ^ parenting skills | Attendance, Utilization of program strategies, Achenbach Child Behavior Checklist-Parent Rating Scale (CBCL) |
| Olds et al. (1997)  [ref 90] | Long-term f/u to RCT | USA / Home | Low SES young unmarried mothers. Most were Caucasian ethnicity. | Children: Newborns. No specific age reported; Parents: M=18.2 years. | 325 mothers | | Intervention by nurses for 9 sessions during pregnancy, 23 sessions (average) between birth and 2 years targeting ˅ child abuse and ‘impact on woman’s life course’. | Mother’s self-report of welfare use and case file data from courts regarding arrests and Child Protection Services regarding abuse. |
| Olds D, Henderson CRJ, Cole R, Eckenrode J, Kitzman H, Luckey D, et al. Long-term effects of nurse home visitation on children's criminal and antisocial behavior: 15-year follow-up of a randomized controlled trial. J Am Med Assoc. 1998;280(14):1238–44. https://doi.org/10.1001/jama.280.14.1238. | Long-term f/u to RCT | USA / Home & Clinic | Young low SES mothers in f/u 15 years after involvement in original study with their children. | Children: M=16.88 weeks; Mother: M=32.96 years. | 315 mothers | | Intervention by nurses for 9 sessions during pregnancy and 23 sessions (average) between birth and 2 years targeting prevention of criminal and anti-social beh. | Self-reports of adult children regarding criminal activity (e.g., arrests, convictions) & sexual beh and substance use). Access to school records regarding academic and beh performance. |
| Ordway, McMahon, Kuhn, & Suchman (2018)  [ref 214] | Qual ethno | USA / Clinic | Mothers of infants and toddlers in outpatient mental health clinic, primarily low SES | Mothers: Children: 0-84 months; | 17 mothers & their children | | **Mothing from the Inside Out (MIO):** 12 weekly sessions aimed at ^ parental reflective functioning among mothers involved in an addiction treatment program | Qualitative measures documenting the implementation process of adapting MIO for a community mental health clinic |
| Osofsky et al. (2007)  [ref 61] | One group pre-post | USA/ Clinic | Low SES parents with MH/PTSD referred from courts/child welfare & children with beh problems. Diverse ethnicity & large group of non-Tx completers. | Children: Range 0-5 years. | 129 dyads | | **Parent-child psychotherapy**: Relationship/attachment-based sessions over 3 years targeting ^ child affect regulation and prevention of child maltreatment. | Ages and Stages Questionnaire (ASQ), BDI, PSI, and videotaped parent-child interactions relationship coded with by third party using the Modified Parent-Relationship Coding Scales. |
| Parent et al. (2011)  [ref 45] | RCT | USA/ Research Setting (University laboratory) | Parents with a range of educational backgrounds. Most were married. | Children: M=4.5 years; Parent: M=38.6 years. | 39 parents (87% were mothers) | | **Parenting the Strong Willed Child**: Parenting group targeting ˅ child beh problems. | ECBI, BSI,  Parenting Convergence Scale (PCS). |
| Paris R, Bolton R, Spielman E. Evaluating a home-based dyadic intervention: changes in postpartum depression, maternal perceptions, and mother-infant interactions. Infant Ment Health J. 2011;32(3):319–38. https://doi.org/10.1002/imhj.20299. | Quasi-exp pre-post | USA/ Home | Mothers with PPD & children. Most of Black ethnicity with diverse SES, married, & highly educated. | Children: M=17 weeks. | 25 dyads | | **Early Connections:** 12-16 weekly sessions of dyadic therapy targeting ^ maternal sensitivity to infant cues and ˅ maternal PPD. | BSI, PSI-SF, Maternal Self-Report Inventory-Short Form (MSI-SF), Postpartum Depression Screening Scale (PDSS), and in-home videotaped parent-child interactions coded with the Coding Interactive Behavior (CIB) Manual. |
| Paschall & Mastergeorge (2017)  [ref 215] | RCT | USA/Clinic | Participants from the Early Head Start Research and Evaluation Project; 36.8% White, 51% male, 24.8% income below poverty. | Children up to 14 months postnatal at baseline | 2,876 mothers | | **Head Start** | Three Big task, Center for Epidemiological Studies Depression Scale (CES-D), PSI/SF, Family Environment Scale |
| Pereira et al. (2014)  [ref 216] | RCT | Portugal/Home | Mothers and their 1- to 4-year-old-children from severely deprived families. | Children: M=28.8 months. | 44 children and mothers | | **Video-feedback Intervention**  **(VIPP-SD):** ˅ harsh discipline ^ parental sensitivity | Observations of a clean-up task and a don’t-touch task; Daily Hassles Questionnaire |
| Phillips et al. (2008)  [ref 156] | Qualitative: Case Study | Australia/ Clinic | Mothers & children at-risk for conduct disorder. Most were married and highly educated. | Children: M=33.8 months. | 52 dyads | | **PCIT:** weekly sessions targeting ˅ child beh problems, ˅ parenting stress and ^ parent mood. | CBCL, ECBI, PSI-SF, DASS and TAI. |
| Pisterman S, McGrath P, Firestone P, Goodman JT, Webster I, Mallory R. Outcome of parent-mediated treatment of preschoolers with attention deficit disorder with hyperactivity. J Consult Clin Psychol. 1989;57(5):628–35. https://doi.org/10.1037/0022-006X.57.5.628. | RCT | USA/ Clinic | Parents of children with ADHD. Most highly educated English speakers & cohabitating, with significant number of single parents. | Children: M=49.8 months. | 50 parent-child dyads (98% of parents were mothers) | | Manualized parenting group and individual sessions with parents & children targeting ˅ child beh problems and ^ parenting skills. | Parent-child interaction observed during free play, a compliance task, and parent-supervised activities. |
| Posthumus et al. (2012)  [ref 132] | Case control design | Netherlands/ Community-based | Parents of conduct difficulties; Demographics: | Children: M=50.11, 51.3 (I, C) months; Parent: M=35.5, 34.1 (I, C) years. | 144 parents | | **IY:** 11 Basic sessions followed by 7 Advanced sessions of a parenting group targeting ˅ child beh problems. | CBCL, and videotaped parent-child interaction task coded with Forehand and McMahon's procedure. |
| Presnall, Webster-Stratton, & Constantino (2014)  [ref 217] | RCT | USA | 280 children 3 to 8 with a family psychiatric history of externalizing behavior | Re-analysis of 5 RCTs |  | | Incredible Years | CBCL |
| Price et al. (2012)  [ref 79] | Long-term f/u to Clustered RCT | Australia/ Clinic | Parents involved in well-baby check with range of educational backgrounds and SES levels. | Children: M=1.6 months; Mothers= 33.1 years. | 225 parents | | Intervention by nurses targeting ˅ child emotional & beh problems and ˅ child sleep problems. | SDQ, Pediatric Quality of Life Inventory (PedsQL), Child-Parent Relationship Scale Short-Form (CPRS-SF) and DASS. |
| Rahman et al. (2008)  [ref 80] | Clustered RCT | Pakistan/ Home | Depressed mothers from rural Pakistan and their children. Diverse SES and education level. | Mothers: M=26.5, 27 (I, C) years. | 903 mothers | | **Thinking Healthy Program:** CBT based intervention by health professional visitors starting in last trimester targeting ˅ maternal depression symptoms | HRSD, Brief Disability Questionnaire (BDQ), and Multidimensional scale for perceived social support (MSPSS). |
| Rapee RM, Jacobs D. The reduction of temperamental risk for anxiety in withdrawn preschoolers: a pilot study. Behav Cogn Psychother. 2002;30(2):211–5. https://doi.org/10.1017/S1352465802002084. | Quasi-exp pre-post with f/u | Australia/ Clinic | Mothers of boys who are inhibited/anxious. | Children: M=56.3 months. | 7 mothers | | Education program for mothers targeting ˅ child anxieties. | Childhood Temperament Questionnaire (CTQ)-Australian Version, Revised Children's Manifest Anxiety Scale (RCMAS). |
| Rapee et al. (2005)  [ref 133] | Quasi-exp pre-post with f/u | Australia/ Clinic | Parents (mothers and fathers) of preschoolers with inhibited traits. Most university educated & Anglo-Saxon background. | Children: M=46.8 months; Mothers: M=35 years; Fathers: M=37.9 years. | 146 parents | | Parent education group targeting ˅ child anxiety. | CTQ. |
| Rapee 2013  [ref 218] | Long-term f/u to RCT | Australia/  Clinic | Adolescents of mother’s involved in early intervention for internalizing disorders 11 years prior | Children were originally 3-5 years |  | **Cool Little Kids**: 6 week group 1.5 hour duration for parents targeting education re: risk for internalizing disorders, reduction of parental overprotection, technique to address child independence | | Spence Children’s Anxiety scale (child & parent versions); Short Moods and Feelings Questionnaire (child & parent version); Automatic Thoughts Scale; Child Anxiety Life Interference Scale; Temperament Assessment Batter for Children. |
| Reynolds, Temple, White et al. (2011)  [ref 91] | Long-term f/u to RCT | USA / School | Low income preschool children who are members of the Chicago Child-Parent Centres | Children: Range 3-4 years during intervention and 26 years at f/u. | 1539 children | **Chicago Child Parent Centres (CPC):** 1-2 year preschool program; 3 hours/day, five days/week with a 6 week summer component targeting ^ school readiness, ^ future occupational prestige, ˅ future arrests and ˅ future depressive symptoms. | | BSI, mother completed surveys for occupational prestige and State & county data accessed related to criminal acts, employment and education. |
| Reynolds & Ou (2011)  [ref 81] | Long-term f/u to quasi-exp | USA / Home & School | Preschool children followed in the Chicago Child Parent Centres. | No specific age data provided. | 1372 children | | **Chicago CPC:** 1-2 year preschool program; 3 hours/day, five days/week with a 6 week summer component targeting ^ school readiness, ^ future occupational prestige, ˅ future arrests and ˅ future depressive symptoms. | BSI, mother completed surveys for occupational prestige and State & county data accessed related to criminal acts, employment and education. |
| Reynolds, Temple, Ou, Robertson et al. (2007)  [ref 92] | Long-term f/u to quasi-exp | USA / Home | Low SES mothers and their children who participated in **Chicago** **Child Parent Centre Early Education Program** (CPC): Preschool for 1-2 years. Most were of black ethnicity and significant number of mothers under 18 (16.4%). | Age related information not provided. | 1400 children | | **Chicago CPC:** 1-2 year preschool program; 3 hours/day, five days/week with a 6 week summer component targeting ^ school readiness, ^ future occupational prestige, ˅ future arrests and ˅ future depressive symptoms. | BSI, mother completed surveys for occupational prestige and State & county data accessed related to criminal acts, employment and education. |
| Roggman, Boyce, & Cook. (2009)  [ref 111] | Repeat measures | USA/ Home | Low SES parents & children in Head Start. Parents recruited were pregnant or with infants under 10 months.  **Status**: Significant number on public assistance, ethnic minorities & teen mothers. | Children: Range 0-36 months; Mother: M=22.84 years. | 161 families | | **Head Start Program:** 36 month parent-focused para-professional intervention targeting ^ child cognitive skills and ^ attachment. | Bayley-III, Attachment Q-Set, and CESD-R. |
| Sadler et al. (2013)  [ref 219] | RCT | USA / Home & clinic | Mothers of lower-income residing in underserviced communities. Diverse cultural background including African American, Puerto Rican, Mexican & Honduran. | Mothers: M=19.7; 19.5 years (I, C); Child: Infant | 105 parents (60 I; 45 C) | | **Minding the Baby:** a manualized multidisciplinary program including home-visitation and infant-parent psychotherapy | CES-D; Brief Symptom Inventory-short form; Parental Bonding Instrument; AMBIANCE; Strange Situation Procedure. |
| Salomonsson & Sandell (2011a)  [ref 170] | RCT | Sweden/ Clinic | Mothers concerned about parent ability & child’s well-being & their children’s beh. | Children: Range 0-1.5 years. | 75 dyads. | | **Mother Infant Psychoanalysis** **(MIP)**: targeting ˅ maternal depression and ^ maternal sensitivity to infant cues. | Questionnaires and independent ratings of mother-child interactions. |
| Salomonsson & Sandell (2011b)  [ref 171] | RCT | Sweden/ Clinic | Mothers concerned about parent ability & child’s well-being & their children. | Children: Range 0-1.5 years. | 75 dyads. | | **MIP**: dyadic sessions targeting ˅ maternal depression and ^ maternal sensitivity to infant cues. | ASQ, EPDS, Satisfaction with Performance Scaled Questionnaire (SPSQ), IR-GAS, and EAS. |
| Salomonsson et al. (2015a)  [ref 220] | Long-term f/u to RCT | Sweden/ Clinic | Mothers concerned about parent ability & child’s well-being & their children’s beh. | Children 0-1.5 years during original study. 3.5 years post treatment. | 66 dyads. | | Compared MIP with Child Health Centre Care. MIP included dyadic session targeting ˅ maternal depression and ^ maternal sensitivity to infant cues. | WPPSI; CGAS; Ages & Stages Questionnaire; Strengths & Difficulties Questionnaire; Story Stem Assessment Profile. |
| Salomonsson et al. (2015b)  [ref 221] | Long term f/u to RCT | Sweden/ Clinic | Mothers concerned about parent ability & child’s well-being & their children’s beh. | Children 0-1.5 years during original study. 3.5 years post treatment. | 66 dyads | | Compared MIP with Child Health Centre Care. MIP included dyadic session targeting ˅ maternal depression and ^ maternal sensitivity to infant cues. | ASQ: CGAS; Working Model of the Child Interview; |
| Sanders et al. (2004)  [ref 118] | RCT | Australia/ Community-based | Parents of children at risk for child abuse; Most were married, lower SES and high school educated. | Children: M=53.71, 52.84 (I, C) months; Mothers: 33.29, 33.68 (I, C) years; Father’s age, 35.32, 36.45 (I, C) years. | 98 parents | | **Positive Parenting Program** (Triple P): parenting group targeting ˅ parental anger, ˅ parental negative cognitions and ˅ risk for child maltreatment. | ECBI, Parent Daily Report (PDR), Home and Community Problem Checklist (HCPC), Parent Attribution for Child Behaviours Measure (PACBM), State Trait Anger Expression Inventory (STAXI-2), PSOC, Parental Anger Inventory, CCAPI, Parent Problem Checklist (PPC) |
| Scheeringa M, Weems C, Cohen J, Amaya-Jackson L, Guthrie D. Trauma-focused cognitive behavioural therapy for posttraumatic stress disorder in three-through six year-old children: a randomized clinical trial. J Child Psychol Psychiatry. 2011;52(8):853–60. https://doi.org/10.1111/j.1469-7610.2010.02354.x. | RCT | USA / Clinic | Preschool children with a diagnosis of PTSD. | Children: M=5.3 years; Mothers: M=34.8 years. | 64 children | **Trauma Focused Cognitive Behavioural Therapy:** child focused therapy, joined by parents for first 2 and last session, observed by other parents targeting ˅ PTSD symptoms, ˅ conduct problems and ˅ depressive symptoms. | | Adverse Events Checklist. |
| Schwarz et al. (2012)  [ref 96] | RCT | USA/ Home | Low SES mothers from high poverty regions and their children. Most were of African American ethnicity. | Children: M=33.6 months; Mothers: M=23.1 years. | 302 mothers | | **MOM Program**: 3 year intervention with mothers targeting ^ use of health services, ^ access to employment resources. | Mother interviewed use of services, and immunization data obtained from primary health clinics. |
| Schweinhart L, Weikart D. Effects of Perry Preschool Program on youths through age 15. J Early Interv. 1980;4(1):29–39. https://doi.org/10.1177/105381518100400105. | RCT | USA/Home  & Preschool | Children from parents with low educational attainment, low occupational status and low cognitive ability | Children:  Range 3-4 years. | 123 children | | **Perry Preschool Project:** child and mother intervention targeting ^ cognitive ability, ^ scholastic achievement, ^ commitment to schooling and ˅ special education in child | IQ tests, school achievement tests, child rating scales, parent and youth interviews, and school records. |
| Shelton TL, Woods JE, Williford AP, Dobbins TR, Neal JM. System of care interventions for hard to manage preschoolers in head start. Research & Training Centre for Children’s Mental Health Annual Conference Proceedings; 2001. | Quasi-exp repeat measures | USA/ Community-based | Parents of preschoolers in Headstart with beh difficulties. Most were of African American ethnicity. | Children: Range 3-4 years. | 28 parents (no gender breakdown given for parents) | | **Project Mastery**: 10 session parenting program including teachers targeting ˅ child beh problems. | BASC-2, ADHD Rating Scale-IV, PSI-SF, PS, PSS, Child Behavior Management Questionnaire, Teacher Strategies Questionnaire (TSQ) and BASC-2 and ECERS-R. |
| Sheridan et al. (2010)  [ref 112] | Quasi-exp pre-post | USA/ Home & School | Low income families on social assistance. Diverse ethnicity & education. | Children: M=43.06 months. | 220 children | | **Getting Ready Program**-parent-school engagement program targeting ^ parental school engagement, ^ parental support of child’s school attendance and ^ parent-child relationship. | Teacher completed SCBE and Devereux Early Childhood Assessment. |
| Sidor et al. (2013)  [ref 222] | QED | Germany/ Home-based | Low income families; mothers with history of mental health issues. | Mothers: M=24.5; 28.2 (I, C) | 302 (150 I; 152 C) | | **Nobody Slips Through the Net:** parent training provided by midwives to ^ skills to parent infants | CARE Index; Ages & Stages Questionnaire; EDPS; PSI-SF. HBS. |
| Siegel E, Bauman KE, Schaefer ES. Hospital and home support during infancy: impact on maternal attachment, child abuse and neglect, and health care utilization. Pediatrics. 1980;66(2):183–90. | RCT | USA/ Home & Hospital-based | Low SES mothers who have just given birth. | Children: Reported only as newborns. Mothers: M=21 years. . | 321 mothers | | Intervention for 5 hours/day during hospitalization and 6-12 month home-based intervention by para-professionals after discharge targeting ˄ maternal attachment and ˅ risk of child abuse. | Case file data regarding maltreatment from Child Protection Services, and medical health record data for health care use. Videotaped parent-child interactions coded with Attachment Inventory. |
| Smyke, et al. (2010)  [ref 163] | RCT | Romania/ Home | Foster-parents & children who were in Romanian orphanages. | Children: M=42.37 months. | 136 children | | Component of the **Bucharest Early Intervention Project:** Social workers supporting foster parents (versus orphanage care) targeting ^ attachment. | SSC, Observation of Care Giving Environment, and Bayley-III. |
| Smyke et al. (2012)  [ref 164] | RCT | Romania/ Home | Foster parents & children from Romanian orphanage. Most children from Roma ethnicity. | Children: Range 6-30 months. | 208 children | | Foster parenting (versus orphanage) targeting ^ child inhibition and ˅ child reactive attachment problems. | Disturbances of Attachment Interview, Bayley-III. |
| Somech & Elizur (2012)  [ref 126] | RCT | Israel/ Clinic | Low SES parents of preschoolers with conduct problems. Most were well-educated mothers from intact families of Israeli decent. | Children: M=48.51, 48.62 (I, C) months; Mother M= 33.28, 33.24 (I, C) years; Father: M= 36.12, 37.22 (I, C) years. | 140 dyads | | **Hitkashrut** (translated meaning “Attachment”) a relationship focused parenting intervention targeting ˅ child beh problems, ^ parenting skills and ˅ parenting stress. | ECBI, SDQ, Antisocial Process Screening Device, Child Behavioral Questionnaire, PSI, Marital Quality Scale, APQ. |
| Sourander et al. (2018)  [ref 223] | RCT with f/u | Finland/ Phone & Web-based | Children with ^ levels of ext. behav. | Children: 4 years of age | 464 families (232 I; | | Finnish Internet Version of the **Strongest Families** Phone-based program: focus on ^ parent skills to ^ positive parent-child relationship, ^ positive child beh. ˅ child neg. beh. | Strengths and Difficulties Questionnaire for child: CBCL; Parenting Scale; DASS-21. |
| Stevens-Simon, Nelligan, & Kelly (2001)  [ref 55] | RCT | USA/ Home | Low SES young pregnant mothers. Ethnically diverse. Most married and on Medicaid. | Mothers: M=17.5 years. | 171 mothers | | **CAMP:** integrated services of health care providers trained (i.e., obstetrics, pediatrics), social work and dietician providing home visits targeting ˅ multiple pregnancies, ˅ risk of child maltreatment, ^ access of health services. | Bayley-III, Home Screening Questionnaire. Case file data accessed regarding maltreatment from Child Protection Services. Medical records data used regarding pregnancy, immunizations, hospitalizations and service use. |
| Suchman et al. (2010)  [ref 70] | RCT | USA/ Clinic | English speaking mothers with addictions & children. | Children: M=2.30, 1.88 (I, C) months; Mothers: 31.3, 28.8 (I, C) years. | 47 dyads | | **Mothers-toddler program** (MTP) vs Parent Educator program. 12 weekly MTP sessions targeting ^ maternal sensitivity to infants. | BDI, Parent Development Interview, Working Model of the Child Interview, and videotaped parent-child interactions coded with NCAST. |
| Suchman NE, DeCoste C, Rosenberger P, McMahon TJ. Attachment-based intervention for substance using mothers: a preliminary test of the proposed mechanisms of change. Infant Ment Health J. 2012;33(4):360–71. https://doi.org/10.1002/imhj.21311. | RCT | USA/ Clinic | Parents with addictions & children. Most were Caucasian & child welfare involved. | Children: M=30.21 months; Mother: M=30.21 years. | 19 dyads | | 12 parenting therapy dyadic session targeting ^ maternal reflective functioning. | BDI, Parent Development Interview, Working Model of the Child Interview, and videotaped parent-child interactions coded with NCAST. |
| Tachibana et al. (2012)  [ref 123] | Clustered RCT | Japan / Home | Pairs of mothers and children attending a private kindergarten in Sendai, Japan. | Children: M=5.13 years; Mothers: M=33.78 years. | 238 dyads | **Mother-Child play activity program:** home-based play activities ˅ parenting stress and ^ child cognitive abilities. | | PSI, Goodenough Draw a person test and SB. |
| van Tueijl C, Leseman PP. Improving mother-child interaction in low-income Turkish-Dutch families: a study of mechanisms mediating improvements resulting from participating in a home-based preschool intervention program. Infant Child Dev. 2004;13(4):323–40. https://doi.org/10.1002/icd.363. | Quasi-exp pre-post | Netherlands / Home | Low SES mothers and fathers and their children. Most speak Turkish as primary language. | Children: M=57 months. | 30 parents | | Parent education by para-professionals 5 days/week over 2 years with 30 weeks of bi-weekly visits by experienced mother/community members targeting ˄ mother-child interactions, ˄ child cognitive and language skills, ˅ maternal cognitive distancing and ˄ socio-emotional support to child. | Videotaped parent-child interaction coded based on neo-Piagetian theory. |
| Vandell (2010)  [ref 7] | Long-term f/u to RCT | USA / Home & School | English speaking mothers and children. Low SES, most mothers had less than high school education, 22% Hispanic. | Children: M=4 years. | 1364 children | | 10 hours of childcare targeting ^ child learning ^ child development, ^ child well-being, prevent ext beh in adolescence, and prevent or reduce adolescent risk taking beh. | Weinberger Adjustment  Inventory (WAI), WJ III ACH, and Observational Record of the Caregiving Environment (ORCE). |
| Wadsby (2012)  [ref 157] | Quasi-exp pre-post | Sweden/ Clinic | Mothers with range of psychosocial risk factors (e.g., alcohol, drug, MH, young mothers) previously in Hadagal Parent-Baby Clinic 8 years prior. | Children: Reported to be under 6 months of age at baseline. | 46 dyads | | **PCIT:** dyadic sessions targeting˅ child beh problems. | CBCL, LEI, Interview Schedule for Social Interaction (ISSI) |
